# Dolosigranulum pigrum Cooperation and Competition in Human Nasal Microbiota

**DOI:** 10.1128/mSphere.00852-20

**Published:** 2020-09-09

**Authors:** Silvio D. Brugger, Sara M. Eslami, Melinda M. Pettigrew, Isabel F. Escapa, Matthew T. Henke, Yong Kong, Katherine P. Lemon

**Affiliations:** a Department of Infectious Diseases and Hospital Epidemiology, University Hospital Zurich, University of Zurich, Zurich, Switzerland; b The Forsyth Institute (Microbiology), Cambridge, Massachusetts, USA; c Department of Oral Medicine, Infection and Immunity, Harvard School of Dental Medicine, Boston, Massachusetts, USA; d Department of Epidemiology of Microbial Diseases, Yale School of Public Health, New Haven, Connecticut, USA; e Alkek Center for Metagenomics & Microbiome Research, Department of Molecular Virology & Microbiology, Baylor College of Medicine, Houston, Texas, USA; f Department of Biological Chemistry and Molecular Pharmacology, Harvard Medical School, Boston, Massachusetts, USA; g Department of Molecular Biophysics and Biochemistry and W.M. Keck Foundation Biotechnology Resource Laboratory, Yale University, New Haven, Connecticut, USA; h Division of Infectious Diseases, Boston Children’s Hospital, Harvard Medical School, Boston, Massachusetts, USA; i Section of Infectious Diseases, Department of Pediatrics, Texas Children’s Hospital and Baylor College of Medicine, Houston, Texas, USA; University of Kentucky

**Keywords:** *Dolosigranulum pigrum*, *Corynebacterium*, *Staphylococcus aureus*, *Streptococcus pneumoniae*, microbe-microbe interactions, interspecies interactions, upper respiratory tract, nasal, microbiota, comparative genomics

## Abstract

Multiple epidemiological studies identify Dolosigranulum pigrum as a candidate beneficial bacterium based on its positive association with health, including negative associations with nasal/nasopharyngeal colonization by the pathogenic species Staphylococcus aureus and Streptococcus pneumoniae. Using a multipronged approach to gain new insights into D. pigrum function, we observed phenotypic interactions and predictions of genomic capacity that support the idea of a role for microbe-microbe interactions involving D. pigrum in shaping the composition of human nasal microbiota. We identified *in vivo* community-level and *in vitro* phenotypic cooperation by specific nasal *Corynebacterium* species. Also, D. pigrum inhibited S. aureus growth *in vitro*, whereas robust inhibition of S. pneumoniae required both D. pigrum and a nasal *Corynebacterium* together. D. pigrum l-lactic acid production was insufficient to account for these inhibitions. Genomic analysis of 11 strains revealed that D. pigrum has a small genome (average 1.86 Mb) and multiple predicted auxotrophies consistent with D. pigrum relying on its human host and on cocolonizing bacteria for key nutrients. Further, the accessory genome of D. pigrum harbored a diverse repertoire of biosynthetic gene clusters, some of which may have a role in microbe-microbe interactions. These new insights into D. pigrum’s functions advance the field from compositional analysis to genomic and phenotypic experimentation on a potentially beneficial bacterial resident of the human upper respiratory tract and lay the foundation for future animal and clinical experiments.

**IMPORTANCE** Staphylococcus aureus and Streptococcus pneumoniae infections cause significant morbidity and mortality in humans. For both, nasal colonization is a risk factor for infection. Studies of nasal microbiota identify Dolosigranulum pigrum as a benign bacterium present when adults are free of S. aureus or when children are free of S. pneumoniae. Here, we validated these *in vivo* associations with functional assays. We found that D. pigrum inhibited S. aureus *in vitro* and, together with a specific nasal *Corynebacterium* species, also inhibited S. pneumoniae. Furthermore, genomic analysis of D. pigrum indicated that it must obtain key nutrients from other nasal bacteria or from humans. These phenotypic interactions support the idea of a role for microbe-microbe interactions in shaping the composition of human nasal microbiota and implicate D. pigrum as a mutualist of humans. These findings support the feasibility of future development of microbe-targeted interventions to reshape nasal microbiota composition to exclude S. aureus and/or S. pneumoniae.

## Abstract (precis)

Staphylococcus aureus and Streptococcus pneumoniae infections cause significant morbidity and mortality in humans. For both, nasal colonization is a risk factor for infection. Studies of nasal microbiota identify Dolosigranulum pigrum as a benign bacterium present when adults are free of S. aureus or when children are free of S. pneumoniae. Here, we validated these *in vivo* associations with functional assays. We found that D. pigrum inhibited S. aureus *in vitro* and, together with a specific nasal *Corynebacterium* species, also inhibited S. pneumoniae. Furthermore, genomic analysis of D. pigrum indicated that it must obtain key nutrients from other nasal bacteria or from humans. These phenotypic interactions support the idea of a role for microbe-microbe interactions in shaping the composition of human nasal microbiota and implicate D. pigrum as a mutualist of humans. These findings support the feasibility of future development of microbe-targeted interventions to reshape nasal microbiota composition to exclude S. aureus and/or S. pneumoniae.

## INTRODUCTION

Colonization of the human nasal passages by Staphylococcus aureus or Streptococcus pneumoniae is a major risk factor for infection by the colonizing bacterium at a distant body site (1–5). Interventions that reduce the prevalence of colonization also reduce the risk of infection and transmission (6, 7). S. aureus and S. pneumoniae are major human pathogens that cause significant morbidity and mortality worldwide (8–11). There are also concerns regarding rising rates of antimicrobial resistance (12) and the potential for long-term effects of antibiotics early in life (13). Thus, efforts have recently focused on the identification of candidate bacteria that confer colonization resistance against S. aureus (14–21) and S. pneumoniae (22–25), with particular urgency for S. aureus in the absence of an effective vaccine.

Dolosigranulum pigrum has emerged in multiple studies of the human upper respiratory tract (URT) microbiota, colonizing with or without *Corynebacterium* species, as potentially beneficial and/or protective against colonization by S. aureus and S. pneumoniae (26–53) (reviewed in references 14, 54, 55, 56, and 57). However, little is known about this Gram-positive, catalase-negative, *Firmicute* bacterium, first described in 1993 (58). Microbiota studies sampling either nostrils or nasopharynx have shown very similar results; therefore, for simplicity, we use “nasal” or “nasal passages” to denote the area inclusive of the nostrils through the nasopharynx. D. pigrum and S. aureus are inversely correlated in adult nasal microbiota (30, 41, 59), whereas, in pediatric nasal microbiota, D. pigrum and members of the genus *Corynebacterium* are overrepresented when S. pneumoniae is absent (26, 33). Moreover, children with D. pigrum colonization of the nasal passages are less likely to have acute otitis media (27, 40) and it has been speculated that D. pigrum-dominated microbiota profiles might be more resistant to invasive pneumococcal disease (46). Furthermore, D. pigrum abundance in the nasal passages is inversely associated with wheezing and respiratory tract infections in infants (28) and an abundance of D. pigrum with *Corynebacterium* in adults provides greater community stability in the face of pneumococcal exposure (51). The intriguing inference from these studies that D. pigrum plays a beneficial role in human nasal microbiota deserves further investigation.

In contrast to the data mentioned above, there are very few reports of D. pigrum in association with human disease (60–68). Its frequent identification in human nasal microbiota (26–53, 69–81) and its rare association with infection are consistent with D. pigrum functioning as a commensal and, possibly, as a mutualist of humans––characteristics that support the idea of its potential for future use as a therapeutic. However, its metabolism and its interplay with other nasal bacteria remain uncharted territory. Using a multipronged approach, we have made significant advances in these areas. First, we identified specific species of candidate bacterial interactors with D. pigrum by analyzing nasal microbiota data sets from adults and children. Second, we used *in vitro* phenotypic assays to show that D. pigrum exhibits distinct interaction phenotypes with nasal *Corynebacterium* species, S. aureus, and S. pneumoniae. Third, on the basis of the genomes of 11 distinct D. pigrum strains, we identify key predicted functions and auxotrophies in its core genome plus a diversity of predicted biosynthetic gene clusters (BCGs) in its accessory genome. This critical shift to phenotypic and genomic experimentation marks a significant advance in understanding D. pigrum, a potentially beneficial member of human nasal microbiota.

## RESULTS

### Individual bacterial species are associated with D. pigrum in the nasal microbiota of both adults and children.

**FIG 1:**
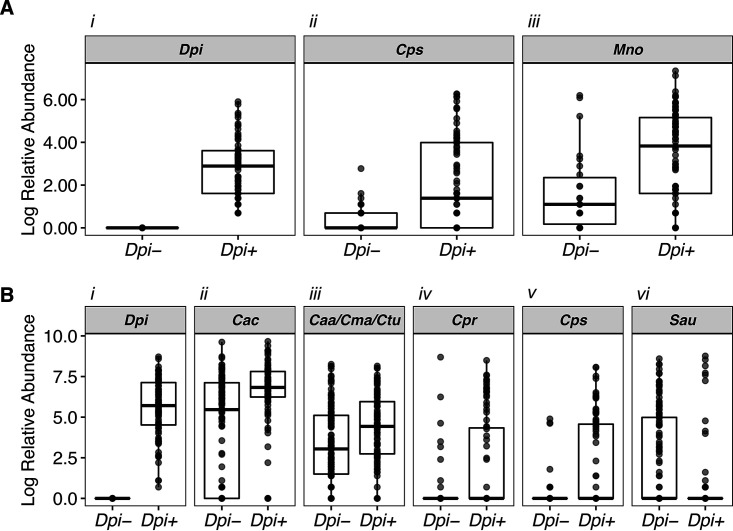
Individual nasal *Corynebacterium* species exhibit increased differential relative abundances in the presence of D. pigrum in human nostril microbiota. We used ANCOM to compare the species/supraspecies-level compositions of 16S rRNA gene nostril data sets from (A) 99 children ages 6 to 78 months and (B) 210 adults where D. pigrum was either absent (*Dpi*−) or present (*Dpi*+) on the basis of 16S rRNA gene sequencing data. Plots show only the taxa identified as statistically significant (sig = 0.05) after correction for multiple testing within ANCOM. The dark bar represents the median; lower and upper hinges correspond to the first and third quartiles. Each gray dot represents the value for a sample, and multiple overlapping dots appear black. *Dpi* = Dolosigranulum pigrum, *Cac* = Corynebacterium accolens, *Caa*/*Cma*/*Ctu* = supraspecies Corynebacterium accolens*_macginleyi_tuberculostearicum*, *Cpr* = Corynebacterium propinquum, *Cps* = Corynebacterium pseudodiphtheriticum, *Mno* = Moraxella nonliquefaciens. Only three species and one supraspecies of *Corynebacterium* from among the larger number of *Corynebacterium* supraspecies/species present in each data set met the significance threshold. Specifically, in the adult nostril data set, there were 21 species and 5 supraspecies groupings of *Corynebacterium* in addition to the reads of *Corynebacterium* that were nonassigned (NA) at the species level. These data were previously published (see Table S7 in reference 41). In the pediatric data set, there were 16 species of *Corynebacterium* in addition to those that were nonassigned among the species-level *Corynebacterium* reads (see Table S2). The Log relative abundance numerical data represented in this figure are available in Table S1.

D. pigrum is the only member of its genus, and multiple 16S rRNA gene-based nasal microbiota studies have identified associations between *Dolosigranulum* and other genera, such as *Corynebacterium* (see, e.g., references 28, 29, 31, 36, 38, 40, 41, 43, and 82). In most cases, the taxonomic resolution in the aforementioned studies was limited to the genus or higher taxonomic levels. Thus, we sought to achieve finer taxonomic resolution and to determine which species are associated with D. pigrum. We identified two nostril data sets with V1-V2/V1-V3 16S rRNA gene sequences, regions that contain sufficient information for species-level taxonomic assignment of most nose-associated bacteria (26, 41). After parsing sequences into species-level phylotypes, we interrogated each data set using analysis of composition of microbiomes (ANCOM) (83) to identify bacterial species that display differential relative abundances in the absence or presence of D. pigrum sequences (Fig. 1; see also Table S1 in the supplemental material). In the nostrils of 99 children ages 6 to 78 months (26), Corynebacterium pseudodiphtheriticum exhibited increased differential relative abundance in the presence of D. pigrum, i.e., was positively associated with D. pigrum, as was Moraxella nonliquefaciens (Fig. 1A). In the nostrils of 210 adults from the Human Microbiome Project (HMP), three *Corynebacterium* species––Corynebacteriu accolens, C. propinquum, and C. pseudodiphtheriticum––and an unresolved supraspecies of C. accolens*-macginleyi-tuberculostearicum* were positively associated with D. pigrum (Fig. 1B, panels ii to v), whereas S. aureus was negatively associated with D. pigrum (Fig. 1B, panel vi). The associations identified in compositional microbiota data observed here and in prior studies (28, 29, 31, 36, 38, 40, 41, 43, 82) led to testable hypotheses about possible direct microbe-microbe interactions between D. pigrum and the specific nasal *Corynebacterium* species, as well as between D. pigrum and S. aureus. Therefore, we used *in vitro* phenotypic assays to test our hypotheses about direct microbe-microbe interactions.

10.1128/mSphere.00852-20.5 TABLE S1 ANCOM Log abundance values. (A) Adult nostril ANCOM data (plotted in Fig. 1A). (B) Pediatric nostril ANCOM data (plotted in Fig. 1B). Download Table S1, XLSX file, 0.02 MB. Copyright © 2020 Brugger et al. 2020 Brugger et al. This content is distributed under the terms of the Creative Commons Attribution 4.0 International license.

### Nasal *Corynebacterium* species can enhance the growth of D. pigrum *in vitro*.

**FIG 2:**
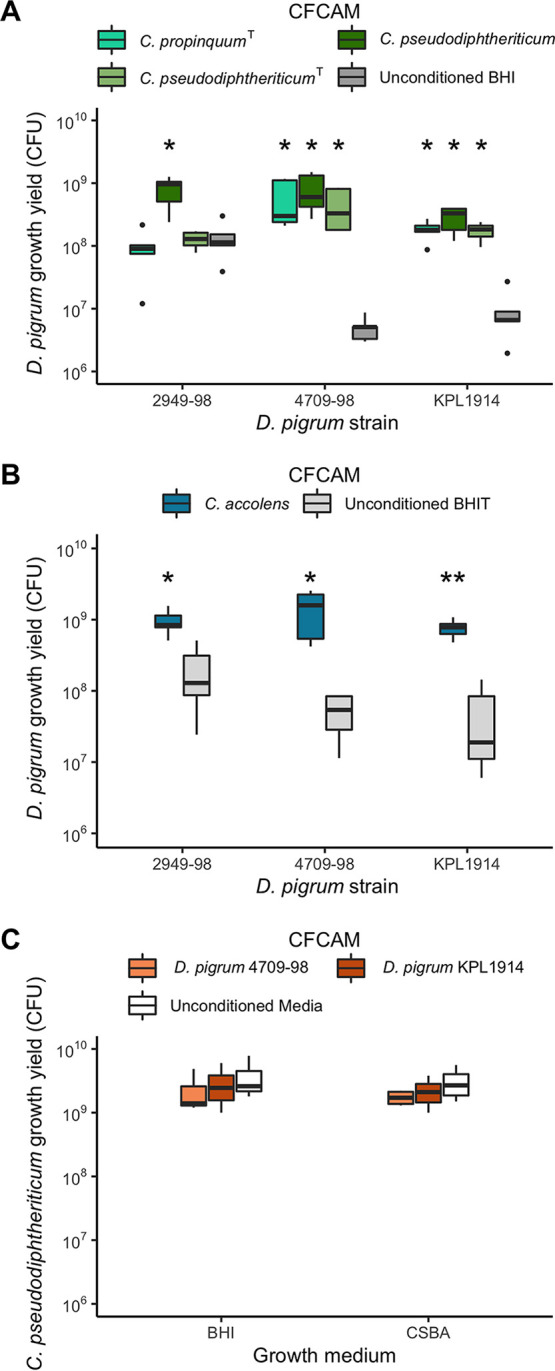
D. pigrum growth yields increase on cell-free conditioned agar medium (CFCAM) from nasal *Corynebacterium* species but not in reverse. (A and B) Growth yield of D. pigrum strains CDC2949-98, CDC4709-98, and KPL1914 was quantified as the number of CFU grown on a polycarbonate membrane placed on (A) cell-free conditioned BHI agar from C. propinquum (aqua green) or C. pseudodiphtheriticum (dark and light green) or (B) cell-free conditioned BHI-triolein (BHIT) agar from C. accolens (blue) and compared to growth on unconditioned BHI agar (dark gray) or unconditioned BHIT agar (light gray), respectively. (C) Growth yield of C. pseudodiphtheriticum KPL1989 on CFCAM from D. pigrum strains (orange) compared to unconditioned medium (white) was assessed similarly. BHIT was used for growth of C. accolens since it is a fatty acid auxotroph and releases needed oleic acid from triolein. Preconditioning strains were grown on a 0.2-μm-pore-size, 47-mm-diameter polycarbonate membrane for 2 days to generate CFCAM. After removal, we then placed a new membrane on the CFCAM onto which we spread 100 μl of target bacterial cells that had been resuspended to an OD_600_ of 0.50 in 1× PBS. After 2 days of growth, CFU were enumerated as described in Materials and Methods. CFU counts were compared independently for each individual strain (A and B, *n* = 5) or medium (C, *n* = 4) using a Wilcoxon rank sum test with Bonferroni correction for multiple comparisons to the unconditioned medium. Dark bars represent medians, lower and upper hinges correspond to the first and third quartiles, and outlier points are displayed individually. *, *P* ≤ 0.05; **, *P* ≤ 0.001. CSBA, citrated sheep blood agar.

We hypothesized that the strong positive association between D. pigrum and the nasal passage-associated *Corynebacterium* species might be due to these *Corynebacterium* species releasing metabolites that enhance the growth of D. pigrum. To test this, we quantified D. pigrum growth yields on unconditioned agar medium compared to the yields seen on cell-free agar medium conditioned by growth of C. pseudodiphtheriticum, C. propinquum, or C. accolens (Fig. 2). Conditioning agar medium by prior growth of any of these three nasal *Corynebacterium* species increased the yield (measured as CFU) of two D. pigrum strains (CDC4709-98 and KPL1914) by 1 to 2 orders of magnitude compared to growth on unconditioned agar medium (Fig. 2A and B). Additionally, one strain of C. pseudodiphtheriticum (Fig. 2A) and the C. accolens strain (Fig. 2B) increased the growth yield of D. pigrum CDC2949-98, a strain with a higher baseline growth yield. The increases in D. pigrum growth yield on *Corynebacterium* cell-free conditioned agar medium (CFCAM) might have resulted from increased growth rate or increased viability or both and might be consistent with the nasal *Corynebacterium* species either removing a toxin from the medium or releasing a metabolite that enhances growth and/or survival of D. pigrum.

In contrast to the increase in D. pigrum growth yield on C. pseudodiphtheriticum CFCAM (Fig. 2A), there was no increase in C. pseudodiphtheriticum strain KPL1989 growth yield on D. pigrum CFCAM (Fig. 2C). Thus, this growth enhancement goes in one direction from nasal *Corynebacterium* species to D. pigrum. This is consistent with unilateral cooperation of nasal *Corynebacterium* species––C. pseudodiphtheriticum, C. propinquum or C. accolens––with D. pigrum in the nostril microbiota and supports the observed positive *in vivo* community-level relationships (Fig. 1).

The positive association between C. accolens and D. pigrum in adult nostril microbiota data sets indicates that *in vivo* positive interactions between C. accolens and D. pigrum prevail (Fig. 1B, panel ii). However, *in vitro*, we observed either a positive or a negative interaction between C. accolens and D. pigrum depending on the assay conditions. C. accolens, unlike C. propinquum and C. pseudodiphtheriticum, is a fatty acid auxotroph, and triolein, a model host epithelial-surface triacylglycerol, served as a source of needed oleic acid in our assays. We observed increased D. pigrum growth yield on a semipermeable membrane atop C. accolens CFCAM consisting of brain heart infusion (BHI) agar supplemented with triolein (BHIT) (Fig. 2B). In contrast, D. pigrum was inhibited when placed directly onto this same C. accolens CFCAM (Table 1). This inhibition is reminiscent of our previous finding that the C. accolens triacylglycerol lipase LipS1 hydrolyzes triacylglycerols, releasing free fatty acids that inhibit S. pneumoniae (33), and S. pneumoniae served as a positive control for the effect of C. accolens in this assay (Table 1). Both D. pigrum and S. pneumoniae belong to the order *Lactobacillales*, and we hypothesized that D. pigrum might be similarly susceptible to free fatty acids such as the oleic acid that C. accolens releases from triolein. Indeed, we observed that oleic acid inhibited D. pigrum when we challenged D. pigrum with oleic acid using a disk diffusion assay with S. pneumoniae as a positive control (Table 2). We also challenged D. pigrum with various concentrations of oleic acid spread onto plates of BHI agar medium. Similarly to the membrane-mediated effect seen in the C. accolens CFCAM experiment described above, we observed D. pigrum growth at higher concentrations of oleic acid when it was placed on a semipermeable membrane atop the oleic acid-coated medium than when it was placed directly on the oleic acid-coated medium (Table 3). This indicates that the membrane provided some protection from inhibition by oleic acid. Overall, these *in vitro* data indicate that C. accolens can both inhibit the growth of D. pigrum by releasing antibacterial-free fatty acids from host triacylglycerols, such as oleic acid from triolein (Tables 1 and 2), and enhance the growth of D. pigrum by releasing an as-yet-unidentified factor(s) (Fig. 2B). Collectively, these results point to a complex set of molecular interactions between these two species.

**TABLE 1:** In contrast to growth on a semipermeable membrane, D. pigrum is inhibited when grown directly on cell-free C. accolens-conditioned BHI agar supplemented with triolein as a source of oleic acid

Conditioning strain	Growth of target strain^*a*^		
	S. pneumoniae 603 (6B)	*D. pigrum* CDC4709-98	*D. pigrum* KPL1914
*C. accolens* KPL1818	0	0	0
*C. propinquum*^T^ DSM44285	0	+	+
C. pseudodiphtheriticum KPL1989	+	+	+

a 0, no growth; +, growth detected, *n *≥ 3.

**TABLE 2:** Oleic acid inhibits *D. pigrum* growth

Oleic acid concn (μg/disc)	ZOI (mm)^*a*^		
	S. pneumoniae 603 (6B)	*D. pigrum* CDC4709-98	*D. pigrum* KPL1914
20	10.3 ± 4.7	12.0 ± 2.9	17.0 ± 2.1
50	22.0 ± 5.4	26.8 ± 4.4	28.4 ± 7.0
100	26.3 ± 6.7	35.8 ± 4.5	39.4 ± 5.0

a Mean zone of inhibition (ZOI) ± standard deviation (SD) produced in a disc diffusion assay. ZOIs were measured as the smallest diameter of inhibited growth, and measurements included disc diameter (6 mm). Results from biological replicates (*n* = 4 for S. pneumoniae, *n* = 5 for D. pigrum) were averaged.

**TABLE 3:** A 0.2-μm-pore-size, 47-mm-diameter polycarbonate membrane provides *D. pigrum* with some protection against inhibition by oleic acid *in vitro*^*a*^

Plated vol of oleic acid (μg)	Result for indicated *D. pigrum* strain			
	Growth directly on agar		Growth on membrane	
	KPL1914	CDC4709-98	KPL1914	CDC4709-98
500	0	0	0	+
50	0	0	+	+
5	+	+	+	+
0.5	+	+	+	+
0.05	+	+	+	+
0 (BHI agar)	+	+	+	+
0 (CSBA)	+	+	+	+

a 0, no growth; +, growth detected; *n* = 3. CSBA, citrated sheep blood agar.

### D. pigrum inhibits S. aureus growth.

**FIG 3:**
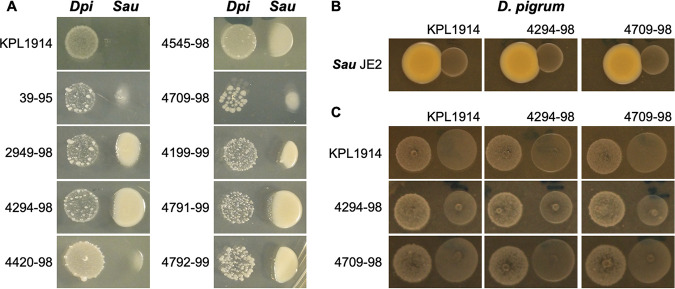
Ten different strains of D. pigrum inhibit methicillin-resistant S. aureus USA300 strain JE2 whereas S. aureus does not inhibit D. pigrum. (A) Ten pregrown D. pigrum isolates produced a diffusible activity that inhibited the growth of S. aureus strain JE2. (B) When S. aureus was pregrown in this assay, there was no visible inhibition of subsequently inoculated D. pigrum. (C) Similarly, we did not observe any inhibition in a pairwise comparison of three representative strains of D. pigrum in this assay. All growth was on BHI agar in the independent experiments (*n *≥ 3) represented in panels A, B, and C. Representative images are shown for each strain. The respective pregrown strain (D. pigrum or S. aureus) was resuspended in PBS, and then a 5-μl drop was placed on BHI agar and pregrown for 48 h (D. pigrum) or 24 h (S. aureus). After that, the indicator strain was inoculated at a location adjacent to the pregrown strain. Inhibition was assessed after 24 and 48 h (48-h results are shown here). For panels A and B, similar results were observed using S. aureus Newman.

The ANCOM of the adult nostril microbiota data set revealed a negative association between S. aureus and D. pigrum (Fig. 1B, panel vi). Direct antagonism would be the simplest mechanism underpinning this observation. Therefore, we assayed for the effect of 10 different strains of D. pigrum on S. aureus. We gave D. pigrum a head start to compensate for its lower growth rate *in vitro.* S. aureus growth was inhibited when it was inoculated adjacent to a pregrown inoculum of each of these 10 D. pigrum strains on agar medium (Fig. 3A). In contrast, a pregrown inoculum of S. aureus did not inhibit D. pigrum (Fig. 3B). Furthermore, comparing three representative D. pigrum strains, none of the D. pigrum strains, when pregrown, inhibited the other D. pigrum strains (Fig. 3C).

### D. pigrum production of lactic acid is unlikely to be the primary mechanism for negative associations with S. pneumoniae or S. aureus.

**FIG 4:**
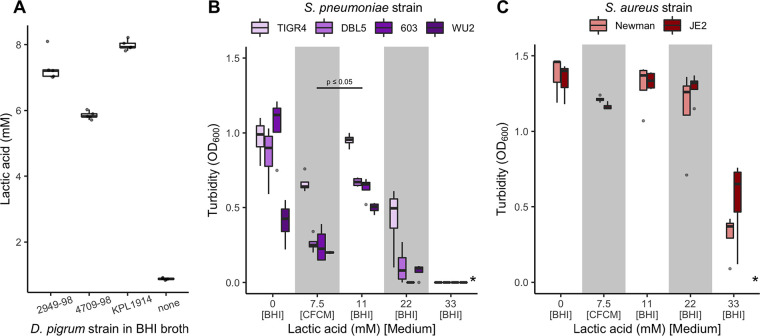
Lactate production by D. pigrum is insufficient to inhibit pathobiont growth. Strains of S. pneumoniae and S. aureus grew in the presence of higher levels of l-lactic acid than those produced by D. pigrum *in vitro*. (A) The concentration of l-lactic acid (mM) produced by three D. pigrum strains was measured after 24 h of gentle shaken aerobic growth in BHI broth at 37°C (*n* = 5) compared to the basal concentration of l-lactic acid in BHI medium alone (none). (B) The average growth (OD_600_) of 4 S. pneumoniae strains in D. pigrum KPL1914 CFCM or in unconditioned BHI broth supplemented with different concentrations of l-lactic acid measured after 19 to 20 h of static aerobic growth at 37°C (*n* = 4). (C) The average growth (OD_600_) of 2 S. aureus strains in D. pigrum KPL1914 CFCM or in unconditioned BHI broth supplemented with different concentrations of l-lactic acid measured after 19 to 20 h of shaken aerobic growth at 37°C (*n* = 4). In panels A and B, the average pH of the D. pigrum CFCM was 6.40 (±0.06). The pH of BHI without lactic acid (0 mM) was adjusted to match the pH of the CFCM, to control for any effect of pH alone. Average levels of growth of S. pneumoniae in CFCM and 11 mM l-lactic acid were analyzed independently for each individual strain using a Wilcoxon rank sum test. Dark bars represent medians, lower and upper hinges correspond to the first and third quartiles, and outlier points are displayed individually, except in panel A, where dots for all individual sample values are represented. *, none of the S. pneumoniae or S. aureus strains displayed growth in 55 mM l-lactate.

D. pigrum lactic acid production has been proposed as a mechanism to explain epidemiologic observations of negative associations between D. pigrum and S. pneumoniae (82). Under nutrient-rich conditions *in vitro*, three tested strains of D. pigrum produced from 5.7 to 8.2 mM l-lactic acid, with strain KPL1914 producing the highest concentration (Fig. 4A). Therefore, we assayed for growth of S. pneumoniae in D. pigrum KPL1914 cell-free conditioned medium (CFCM) and in BHI broth supplemented with various concentrations of l-lactic acid. Three of the four S. pneumoniae strains tested showed some growth in 22 mM lactic acid (Fig. 4B), and all strains displayed more growth in BHI medium supplemented with 11 mM l-lactic acid than in the D. pigrum KPL1914 CFCM, which had 7.5 mM D. pigrum-produced l-lactic acid (Fig. 4B). Furthermore, growth of S. pneumoniae alone under these conditions resulted in a higher concentration of l-lactic acid than did D. pigrum growth (see Fig. S1 in the supplemental material). Thus, the restriction of S. pneumoniae growth in D. pigrum CFCM is unlikely to have been due to production of lactic acid by D. pigrum. More likely, it reflected competition for nutrients since fresh medium was not added to the CFCM, which, therefore, would have a lower concentration of sugars than BHI broth. However, the possibility of D. pigrum production of a toxin and/or an antipneumococcal compound in BHI broth cannot be excluded. We also tested the *in vitro* effect of l-lactic acid on two strains of S. aureus. Both showed some growth in 33 mM lactic acid (Fig. 4C). Thus, D. pigrum did not produce enough l-lactic acid to restrict S. aureus growth under the tested conditions. Furthermore, growth of S. aureus alone resulted in a concentration of l-lactic acid similar to that seen with D. pigrum growth (Fig. S1). In contrast to the S. pneumoniae results, we would not expect depletion of sugars to have a large effect on S. aureus growth in D. pigrum CFCM given its ability to utilize a broader repertoire of energy sources, e.g., amino acids. Indeed, both S. aureus strains showed only a minimal decrease in growth in D. pigrum CFCM. This also revealed differences in D. pigrum production of the anti-S. aureus activity during growth on BHI agar medium (Fig. 3) versus growth in BHI broth (Fig. 4C). Levels of excretion of metabolites may differ during growth in liquid versus on agar medium, and the mechanism of the D. pigrum anti-S. aureus activity is yet to be identified.

10.1128/mSphere.00852-20.1 FIG S1 S. pneumoniae produces higher levels of l-lactic acid than S. aureus, D. pigrum, and C. pseudodiphtheriticum. The concentration (mM) of l-lactic acid produced by the assayed strains was measured after 24 h of aerobic growth with gentle shaking (∼50 to ∼60 rpm) in BHI broth at 37°C (*n* ≥ 4) compared to the basal concentration of l-lactic acid alone in BHI broth (none). Strains were grown and lactic acid concentrations determined as described in Materials and Methods for Fig. 4A with the following differences: (i) optical density was measured in a 96-well format for the lactate assay using a plate reader and (ii) we generated a standard curve of lactate concentrations using measurements from the plate reader to calculate the concentrations of lactate in the CFCM for each strain. *Spn*, S. pneumoniae 603; *Sau*, S. aureus JE2; *Dpi*, D. pigrum KPL1914; *Cpsd*, C. pseudodiphtheriticum KPL1989; none, BHI medium alone. Download FIG S1, PDF file, 0.04 MB. Copyright © 2020 Brugger et al. 2020 Brugger et al. This content is distributed under the terms of the Creative Commons Attribution 4.0 International license.

### D. pigrum and C. pseudodiphtheriticum inhibit S. pneumoniae growth together but not alone.

**FIG 5:**
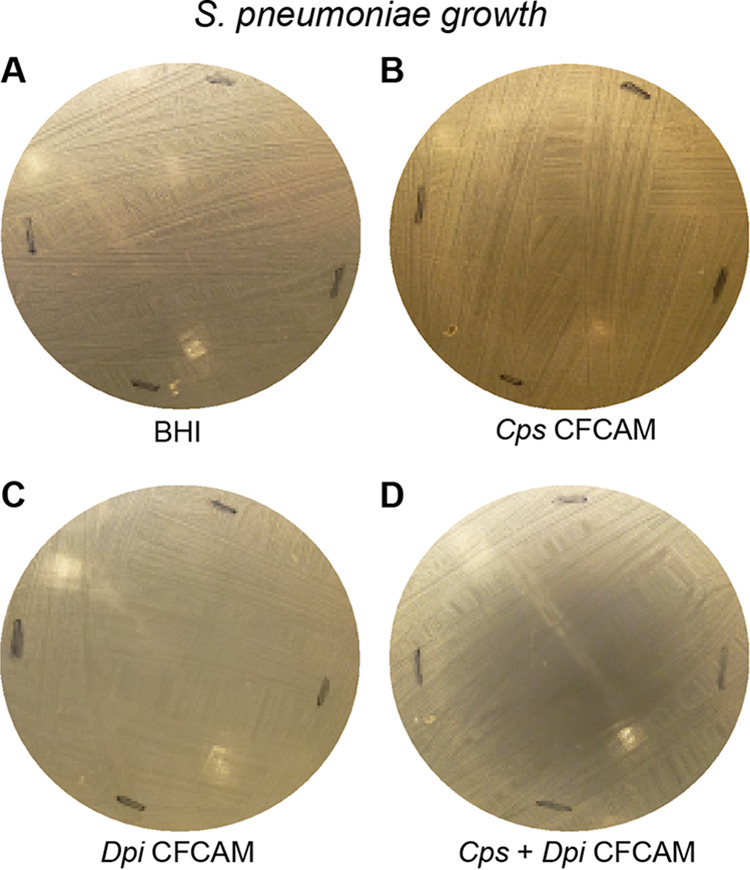
D. pigrum and C. pseudodiphtheriticum grown together but not D. pigrum alone inhibited S. pneumoniae in an *in vitro* agar medium-based assay. Representative images are shown of S. pneumoniae 603 growth on (A) BHI medium alone or on CFCAM from (B) C. pseudodiphtheriticum KPL1989, (C) D. pigrum KPL1914, or (D) both D. pigrum and C. pseudodiphtheriticum grown in a mixed inoculum (*n* = 4). To condition the medium, we cultivated D. pigrum and/or C. pseudodiphtheriticum on a membrane, which was then removed prior to spreading a lawn of S. pneumoniae. For monoculture, 100 μl of either D. pigrum or C. pseudodiphtheriticum, resuspended to an OD_600_ = 0.50, were inoculated onto the membrane. For mixed coculture, 50 μl of D. pigrum (OD_600_ = 0.50) were mixed with 50 μl of C. pseudodiphtheriticum (OD_600_ = 0.50) to yield a final volume of 100 μl for the inoculum, such that each bacterial species was present in the coculture inoculum at half the amount used for the corresponding monoculture inoculum. Images were cropped. Black marks indicate edges where the membrane had been.

Since the effects seen with C. pseudodiphtheriticum were positively associated with the presence of D. pigrum (Fig. 1), we investigated their combined effects on S. pneumoniae growth. Agar medium conditioned with a coculture of C. pseudodiphtheriticum strain KPL1989 and D. pigrum strain CDC4709-98 inhibited S. pneumoniae growth, whereas agar medium conditioned with a monoculture of either C. pseudodiphtheriticum or D. pigrum alone did not (Fig. 5; see also Fig. S2). This might have been due to cocultivation resulting either in a greater level of nutrient competition than monoculture of either commensal alone or in the production of a diffusible compound(s) toxic/inhibitory to S. pneumoniae by either D. pigrum or C. pseudodiphtheriticum or both D. pigrum and C. pseudodiphtheriticum when grown together. (Of note, cocultivation of D. pigrum and C. pseudodiphtheriticum in liquid BHI medium did not increase the level of l-lactic acid compared to growth of D. pigrum alone [Fig. S1].) Along with the *Corynebacterium* species enhancement of D. pigrum growth yield (Fig. 2) and the D. pigrum inhibition of S. aureus growth (Fig. 3), these data indicate that the negative associations of D. pigrum with S. aureus and S. pneumoniae are likely mediated by different molecular mechanisms.

10.1128/mSphere.00852-20.2 FIG S2 Grown together, D. pigrum and C. pseudodiphtheriticum inhibit S. pneumoniae. A second set of representative images of S. pneumoniae 603 growth on (A) BHI medium alone or on CFCAM in the presence of (B) C. pseudodiphtheriticum KPL1989, (C) D. pigrum KPL1914, or (D) both D. pigrum and C. pseudodiphtheriticum in a mixed inoculum (*n* = 4) is shown. To condition the medium, we cultivated D. pigrum and/or C. pseudodiphtheriticum on a membrane, which was then removed prior to spreading a lawn of S. pneumoniae. Images were cropped. Black marks indicate edges of regions where the membrane had been. Download FIG S2, PDF file, 0.1 MB. Copyright © 2020 Brugger et al. 2020 Brugger et al. This content is distributed under the terms of the Creative Commons Attribution 4.0 International license.

To learn more about the functional capacity and genomic structure of D. pigrum, we next turned to genomic analysis, which provided insights into some of the epidemiologic and phenotypic observations presented above.

### The genomes of 11 D. pigrum strains reveal a small genome consistent with a highly host-adapted bacterium.

We analyzed one publicly available genome of D. pigrum (ATCC 51524) and sequenced 10 additional strains (see Text S1 in the supplemental material), which were selected to ensure representation of distinct strains (see Materials and Methods). To start, we focused on basic genomic characteristics. The 11 D. pigrum strain genomes had an average size of 1.86 Mb (median 1.88 Mb) with 1,693 predicted coding sequences (CDS) (see Tables A and B in Text S1). Approximately 1,200 CDS were core (see Fig. A and B and Table C in Text S1) and exhibited a high degree of nucleotide and amino acid sequence conservation (see Fig. C in Text S1). As shown in Text S1, we further analyzed synteny of two closed genomes (see Fig. D in Text S1) and BLAST ring comparisons (see Fig. E in Text S1) and constructed a core-gene-based phylogeny (see Fig. F in Text S1).

10.1128/mSphere.00852-20.3 TEXT S1 Supplemental genomic structure analysis. Download Text S1, PDF file, 2.5 MB. Copyright © 2020 Brugger et al. 2020 Brugger et al. This content is distributed under the terms of the Creative Commons Attribution 4.0 International license.

### D. pigrum is a predicted auxotroph for amino acids, polyamines, and enzymatic cofactors.

The nasal environment is low in and/or lacking in key nutrients such as methionine (84), and the small genome size (1.86 Mb) of D. pigrum is consistent with reduced biosynthetic capacity. To gain insight into how D. pigrum functions in the nasal environment, we examined all 11 genomes, finding evidence of auxotrophy for some amino acids (e.g., methionine), polyamines (e.g., putrescine and spermidine), and enzymatic cofactors (e.g., biotin) across all strains. In turn, we identified putative degradation pathways (e.g., methionine), transporter pathways (e.g., polyamines and biotin), and salvage pathways (e.g., folate), suggesting that D. pigrum acquires some required nutrients exogenously. Section I in Text S2 contains additional details plus predictions of acquisition of metal cofactors. The auxotrophy predictions may be incomplete, since we were unable to grow D. pigrum in a chemically defined medium with all 20 amino acids that was putatively replete on the basis of these predictions. Apparent auxotrophy for a number of required nutrients indicates that these must be available either from the host or from neighboring microbes in human nasal passages, e.g., possibly from nasal *Corynebacterium* species.

10.1128/mSphere.00852-20.4 TEXT S2 Supplemental genomic functional predictions. Download Text S2, PDF file, 0.8 MB. Copyright © 2020 Brugger et al. 2020 Brugger et al. This content is distributed under the terms of the Creative Commons Attribution 4.0 International license.

### Whole-genome sequencing indicates that D. pigrum metabolizes carbohydrates to lactic acid via homofermentation.

D. pigrum produced lactate during *in vitro* cultivation (Fig. 4A). Lactic acid bacteria mainly perform either homofermentation or heterofermentation of carbohydrates (85). Therefore, we examined the genomic capacity of D. pigrum for carbohydrate metabolism (see section II in Text S2). D. pigrum genomes lacked genes required for a complete tricarboxylic acid cycle, which is consistent with fermentation. Moreover, we identified genes encoding a complete glycolytic pathway in all 11 strains that are consistent with homofermentation. All 11 strains harbored a predicted l-lactate dehydrogenase (EC 1.1.1.27) which catalyzes the reduction of pyruvate to lactate, regenerating NAD^+^ for glycolysis (GAPDH [glyceraldehyde-3-phosphate dehydrogenase] step), consistent with homofermentation to l-lactate as the main product of glycolysis.

### The accessory genome of 11 D. pigrum strains contains a diversity of biosynthetic gene clusters predicted to encode antibiotics.

Lactic acid production alone appears insufficient to account for the negative *in vitro* associations of D. pigrum with S. aureus and with S. pneumoniae (Fig. 4). To delve further into the genetic capacity of D. pigrum for possible mechanisms of inhibition, we explored the accessory genome of the 11 sequenced strains. Consistent with a prior report (60), D. pigrum appears to be broadly susceptible to antibiotics (see section III in Text S2). What emerged in our analysis was a diversity of biosynthetic gene clusters (BGCs) (see Table A and Fig. A in Text S2), including a diversity of BGCs predicted to encode candidate antibiotics. Strikingly, although 10 of 10 strains tested displayed inhibition of S. aureus growth *in vitro* (Fig. 3), there was no single BGC common to all 10 strains that might encode a compound with antibiotic activity. On the basis of these data, we hypothesize that D. pigrum uses a diverse repertoire of BGCs to produce bioactive molecules that play key roles in interspecies interactions with its microbial neighbors, e.g., for niche competition, and potentially with its host. This points to a new direction for future research on the functions that underlie the positive associations of D. pigrum in human nasal microbiota with health and highlights the need to develop a system for genetic engineering of D. pigrum.

## DISCUSSION

D. pigrum was shown to be associated with health in multiple genus-level compositional studies of human URT/nasal passage microbiota. In nasal passage microbiota data sets, we identified positive associations of D. pigrum with specific species of *Corynebacterium* in adults and children and a negative association of D. pigrum with S. aureus in adults (Fig. 1). We observed phenotypic support for these associations. First, unilateral cooperation from three common nasal *Corynebacterium* species enhanced D. pigrum growth yields (Fig. 2). Second, D. pigrum inhibited S. aureus (Fig. 3). Our genomic analysis revealed auxotrophies consistent with D. pigrum reliance on cocolonizing microbes and/or the human host for key nutrients. Genomic analysis also showed an aerotolerant anaerobe that performs homofermentation to lactate. However, D. pigrum lactate production (Fig. 4A) was insufficient to inhibit either S. pneumoniae (Fig. 4B) or S. aureus (Fig. 4C) and therefore is not the sole contributor to negative associations with S. pneumoniae and S. aureus *in vivo*. Consistent with the reports of a negative association between D. pigrum (usually in conjunction with *Corynebacterium*) and S. pneumoniae, we observed that cocultivation of D. pigrum and C. pseudodiphtheriticum produced a diffusible activity that robustly inhibited S. pneumoniae (Fig. 5; see also Fig. S2 in the supplemental material) whereas monoculture of either did not. Finally, we uncovered a surprisingly diverse repertoire of BGCs in 11 D. pigrum strains, revealing potential mechanisms for niche competition that were previously unrecognized. These data mark a significant advance in the study of D. pigrum and set the stage for future research on molecular mechanisms.

The *in vitro* interactions of D. pigrum with S. aureus and with S. pneumoniae support inferences from composition-level microbiota data of competition between D. pigrum and each pathobiont. However, these interactions differed *in vitro*. D. pigrum alone inhibited S. aureus, but D. pigrum plus C. pseudodiphtheriticum, together, robustly inhibited S. pneumoniae. These results point to a more complex set of interactions among these specific bacterial members of the human nasal microbiota which likely exists in the context of a network of both microbe-microbe and microbe-host interactions. To date, mechanisms for only a few such interactions have been described. For example, a C. accolens triacylglycerol lipase (LipS1) releases antipneumococcal free fatty acids from model host surface triacyclglycerols *in vitro*, pointing to habitat modification as a possible contributor to S. pneumoniae colonization resistance (33).

Multiple mechanisms could result in D. pigrum inhibition of S. aureus *in vitro*, including nutrient competition and excretion of a toxic primary metabolite or of an anti-S. aureus secondary metabolite (i.e., an antibiotic). Initial bioassay-guided fractionation approaches failed to identify such a mechanism. However, the data showing the existence of diverse repertoires of BGCs among the 11 D. pigrum strains are intriguing because they include predicted bacteriocins, including lanthipeptides. For example, 4 of the 11 strains harbored putative type II lanthipeptide biosynthetic gene clusters. These clusters are characterized by the presence of the LanM enzyme, containing both dehydration and cyclization domains needed for lanthipeptide biosynthesis (86). Alignment of these enzymes with the enterococcal cytolysin LanM revealed conserved catalytic residues in both domains (87). Cleavage of the leader portion of the lanthipeptide is necessary to produce an active compound, and the presence of peptidases and transporters within these BGCs suggests that these D. pigrum strains might secrete an active lanthipeptide, which could play a role in niche competition with other microbes. Additionally, 8 of the 11 D. pigrum genomes examined contain putative bacteriocins, or bactericidal proteins and peptides. Intriguingly, the D. pigrum strains exhibiting the strongest inhibition of S. aureus (CDC4709-98, CDC39-95, and KPL1914) (Fig. 3) were the only strains that contained both a lanthipeptide BGC and a bacteriocin, further indicating that D. pigrum may employ multiple mechanisms to inhibit S. aureus growth. Also, if both are required for the *in vitro* inhibition, this might explain the negative results from bioassay-guided fractionation. Mechanisms are coming to light that account for how other nasal bacteria interact with S. aureus. For example, commensal *Corynebacterium* species excrete a yet-to-be-identified substance that inhibits S. aureus autoinducing peptides blocking *agr* quorum sensing (QS), and shifting S. aureus toward a commensal phenotype (88). Also, the yet-to-be-identified mechanism of C. pseudodiphtheriticum contact-dependent inhibition of S. aureus is mediated through phenol-soluble modulins, the expression of which increases during activation of *agr* QS (89). Within broader *Staphylococcus-Corynebacterium* interactions, C. propinquum outcompetes coagulase-negative *Staphylococcus*, but not S. aureus, for iron *in vitro* using the siderophore dehydroxynocardamine, the genes for which are transcribed *in vivo* in human nostrils (90). Interphylum *Actinobacteria*-*Firmicutes* interactions also occur between Cutibacterium acnes and *Staphylococcus* species (reviewed in reference 14). For example, some strains of C. acnes produce an antistaphylococcus thiopeptide, cutimycin, *in vivo* and the presence of the cutimycin BGC is correlated with microbiota composition at the level of the individual human hair follicle (91). Of note, *Actinobacteria* competition with coagulase-negative *Staphylococcus* species could also have network-mediated (indirect) effects on S. aureus via the well-known competition among *Staphylococcus* species (reviewed in reference 92), which can be mediated by, e.g., antibiotic production (15–17, 19), interference with S. aureus *agr* QS (18, 20, 93, 94), or extracellular protease activity (95), among other means (14). Further rounding out the emerging complexity of microbe-microbe interactions in nasal microbiota, multiple strains of *Staphylococcus*, particularly S. epidermidis, inhibit the *in vitro* growth of other nasal and skin bacteria, including D. pigrum, via yet-to-be-identified mechanisms (16). The evidence described above points to a wealth of opportunity to use human nasal microbiota as a model system to learn how bacteria use competition to shape their community.

Direct cooperation could contribute to the observed positive associations between bacterial species in epidemiological microbiome studies. Conditioning medium with any of the three nasal *Corynebacterium* species positively associated with D. pigrum *in vivo* in human nasal microbiota (Fig. 1) enhanced the growth yield of some D. pigrum strains (Fig. 2). This is possibly accounted for by excretion of a limiting nutrient or by removal of a toxic medium component. The genomic predictions of auxotrophy might favor nasal *Corynebacterium* species providing cooperation to D. pigrum by excretion of a limiting nutrient. Indeed, mass spectrometry indicated that a number of nutrients are limiting in the nose (84).

There were several limitations of our study. First, we analyzed the genomes of 11 strains that were primarily isolated in the setting of disease. It is unclear whether these strains were contaminants or pathogenic contributors (60). However, D. pigrum strains are infrequently associated with disease (61–68). These 11 D. pigrum strains did not appear to encode potential virulence factors (see section III in Text S2 in the supplemental material), which is consistent with D. pigrum acting primarily as a mutualistic species of humans. Second, the ongoing search for a fully defined chemical medium permissive for D. pigrum growth precluded experimental verification of predicted auxotrophies and further investigation of how the presence of nasal *Corynebacterium* enhances D. pigrum growth yields. Third, the D. pigrum anti-S. aureus factor has eluded purification and identification efforts with standard chemistry approaches and D. pigrum is not yet genetically tractable, limiting the use of genetic approaches to identify it. Fourth, to date, there has been no animal model for nasal colonization with D. pigrum and *Corynebacterium* species, which stymies direct *in vivo* testing of the hypothesis of pathobiont inhibition and points to another area of need within the nasal microbiome field.

In summary, we validated *in vivo* associations from human bacterial microbiota studies with functional assays that support the hypothesis that D. pigrum is a mutualist with respect to its human host, rather than a purely commensal bacterium. Further, the data from these phenotypic interactions support the idea of a role for microbe-microbe interactions in shaping the composition of human nasal microbiota and, thus, the possibility of developing microbe-targeted interventions to reshape community composition. The next step will be to identify the molecular mechanisms of those interactions and to assess their role in the human host. Such work could establish the premise for future studies to investigate the therapeutic potential of D. pigrum as a topical nasal probiotic for use in patients with recurrent infections with S. pneumoniae, possibly in conjunction with a nasal *Corynebacterium* species, or with S. aureus, in conjunction with established S. aureus decolonization techniques (96).

## MATERIALS AND METHODS

### Species-level reanalysis of a pediatric nostril microbiota data set.

Laufer et al. analyzed nostril swabs collected from 108 children ages 6 to 78 months (26). Of these, 44% were culture positive for S. pneumoniae and 23% were diagnosed with otitis media. 16S rRNA gene V1-V2 sequences were generated using Roche/454 with primers 27F and 338R. We obtained 184,685 sequences from the authors, of which 94% included sequence matching primer 338R and 1% included sequence matching primer 27F. We performed demultiplexing in QIIME (97) (split_libraries.py), filtering reads for those ≥250 bp in length, those with a quality score of ≥30, and those with barcode type hamming_8. Then, we eliminated sequences from samples for which there were no metadata (*n* = 108 for metadata), leaving 120,963 sequences on which we performed *de novo* chimera removal in QIIME (USEARCH 6.1) (98, 99), yielding 120,274 16S rRNA V1-V2 sequences. We then aligned the 120,274 chimera-cleaned reads in QIIIME (PyNAST) (100), using eHOMDv15.04 (41) as a reference database, and trimmed the reads using “o-trim-uninformative-columns-from-alignment” and “o-smart-trim” scripts (101). A total of 116,620 reads (97% of the chimera-cleaned reads) were recovered after the alignment and trimming steps. After these initial cleaning steps, we retained only the 99 samples with more than 250 reads. We analyzed these data set of 99 samples with a total of 114,909 reads using MED (101) with minimum substantive abundance of an oligotype (-M) equal to 4 and maximum variation allowed in each node (-V) equal to 6 nucleotides (nt), which equals 1.6% of the 379-nucleotide length of the trimmed alignment. Of the 114,909 sequences, 82.8% (95,164) passed the -M and -V filtering and are represented in the MED output. Oligotypes were assigned taxonomy in R with the dada2::assignTaxonomy() function (an implementation of the naive Bayesian RDP classifier algorithm with a kmer size of 8 and a bootstrap value of 100) (102, 103) using the eHOMDv15.1 V1-V3 Training Set (version 1) (41) and a bootstrap value of 70. We then collapsed oligotypes within the same species/supraspecies, yielding the data shown in Table S2 in the supplemental material.

10.1128/mSphere.00852-20.6 TABLE S2 Species-level reanalysis of a pediatric nostril microbiota dataset (related to Fig. 1B). Download Table S2, XLSX file, 0.1 MB. Copyright © 2020 Brugger et al. 2020 Brugger et al. This content is distributed under the terms of the Creative Commons Attribution 4.0 International license.

### Microbiota community comparison (Fig. 1).

The pediatric 16S rRNA gene V1-V2 data set analyzed at the species level (Table S2) and the HMP adult 16S rRNA gene V1-V3 data set previously analyzed at the species level (see Table S7 in reference 41) were used as input for the ANCOM, including all identified taxa (i.e., we did not remove taxa with low relative abundance). ANCOM (version 1.1.3) was performed using the presence or absence of D. pigrum, on the basis of the 16S rRNA gene sequencing data, as group definer. ANCOM default parameters were used (sig = 0.05, tau = 0.02, theta = 0.1, repeated = FALSE [i.e., Kruskal-Wallis test]), except that we performed a correction for multiple comparisons (multcorr = 2) instead of using the default no correction (multcorr = 3) (83). The Log relative abundance values for the taxa identified as statistically significant (sig = 0.05) are represented in Fig. 1 and are also available in Table S1.

### Cultivation from frozen stocks.

Bacterial strains (see Table A in Text S1 in the supplemental material; see also Table S3) were cultivated as described here unless stated otherwise. Across the various methods, strains were grown at 37°C with 5% CO_2_ unless otherwise noted. D. pigrum strains were cultivated from frozen stocks on BBL Columbia colistin-nalidixic acid (CNA) agar with 5% sheep blood (BD Diagnostics) for 2 days. *Corynebacterium* species were cultivated from frozen stocks on BHI agar (C. pseudodiphtheriticum and C. propinquum) or on BHI agar supplemented with 1% Tween 80 (C. accolens) for 1 day. The resuspensions described below were made by harvesting colonies from agar medium and resuspending in 1× phosphate-buffered saline (PBS). Of note, we primarily use agar medium because in our experience D. pigrum exhibits more consistent growth on agar medium than in liquid medium. Likewise, growth on a semisolid surface is likely to better represent growth on nasal surfaces than would growth under the well-mixed conditions of shaking liquid medium.

10.1128/mSphere.00852-20.7 TABLE S3 Non-D. pigrum bacterial strains used in this study. Download Table S3, DOCX file, 0.03 MB. Copyright © 2020 Brugger et al. 2020 Brugger et al. This content is distributed under the terms of the Creative Commons Attribution 4.0 International license.

### Preconditioning growth yield assays (Fig. 2).

To assess the growth yield of D. pigrum on a polycarbonate membrane atop media conditioned by *Corynebacterium* spp., each *Corynebacterium* strain was resuspended from growth on agar medium to an optical density at 600 nm (OD_600_) of 0.50 in 1× PBS. Then, 100 μl volumes of each resuspension were individually spread onto a 0.2-μm-pore-size, 47-mm-diameter polycarbonate membrane (EMD Millipore, Billerica, MA) atop 20 ml of either BHI agar for C. pseudodiphtheriticum and C. propinquum or BHI agar supplemented with triolein (BHIT) (CAS catalog no. 122-32-7, Acros) spread atop the agar medium, as previously described (33), for C. accolens. After 2 days of growth, the membranes with *Corynebacterium* cells were removed, leaving CFCAM. On each plate of CFCAM, we placed a new membrane onto which we spread 100 μl of D. pigrum cells that had been resuspended to an OD_600_ of 0.50 in 1× PBS. After 2 days, the membranes with D. pigrum were removed, placed in 3 ml 1× PBS, and subjected to vortex mixing for 1 min to resuspend cells. Resuspensions were diluted 1:10 six times, dilutions were inoculated onto BBL CNA agar with 5% sheep blood, and CFU were enumerated after 2 to 3 days of growth. To assess the growth yield of C. pseudodiphtheriticum on a polycarbonate membrane atop media conditioned by D. pigrum, strains KPL1914 and CDC4709-98 were grown for 2 days as described above. C. pseudodiphtheriticum KPL1989 growth yield was then measured as described above.

### Growth of D. pigrum directly on BHI agar medium supplemented with triolein and conditioned by growth of nasal *Corynebacterium* species (Table 1).

Onto BHI agar supplemented with 200 U/ml of bovine liver catalase (C40-500MG, Sigma) (BHIC), we spread 50 μl of 100 mg/ml of triolein (BHICT). We then spread 50 μl of a resuspension (OD_600_ of 0.50) of each *Corynebacterium* strain onto a 0.2-μm-pore-size, 47-mm-diameter polycarbonate membrane placed atop 10 ml of BHICT agar in a 100-mm-by-15-mm petri dish. After 2 days, we removed each membrane with *Corynebacterium* cells, leaving CFCAM. Using a sterile cotton swab, we then spread a lawn of either D. pigrum (from cells resuspended to an OD_600_ of 0.50 in 1× PBS) or S. pneumoniae (taken directly from agar medium) onto the CFCAM. Each lawn was then grown for 1 to 2 days before documentation of growth or inhibition of growth was performed with digital photography.

### Oleic acid disc diffusion assay (Table 2).

A lawn of D. pigrum or S. pneumoniae was spread onto 10 ml of BHIC agar using a sterile cotton swab as described above. Oleic acid (Sigma-Aldrich) was dissolved to reach final concentrations of 2 mg/ml, 5 mg/ml, and 10 mg/ml in ethanol, and then we added 10 μl of each to separate, sterile 0.2-μm-pore-size, 6-mm-diameter filter discs (Whatman), with 10 μl of ethanol alone added to a disc as a control. After the solvent was allowed to evaporate, filter discs were placed on the bacterial lawns, which were then allowed to grow for 1 day before measurement of zones of inhibition and photography were performed.

### Growth of D. pigrum directly on versus atop a membrane on oleic acid-coated agar medium (Table 3).

Oleic acid was dissolved in 100% ethanol to a concentration of 5 mg/ml and then further diluted 10-fold 5 times in ethanol. For each dilution, 100 μl was spread on a separate plate of BHI agar medium. Next, 10 μl of D. pigrum KPL1914 and CDC4709-98, each resuspended to an OD_600_ = 0.3, were inoculated both directly onto the oleic acid-coated agar medium and atop of a 0.2-μm-pore-size, 47-mm-diameter polycarbonate membrane (EMD Millipore, Billerica, MA) on the same plate. After the plates had been maintained for 2 days at 37°C, we assessed and photographed the growth. In addition, for each dilution and strain, one spot on the membrane was resuspended in PBS to assess CFU counts after serial dilutions and plating on blood agar plates (see above).

### D. pigrum*-*S. aureus side-by-side coculture assays (Fig. 3).

D. pigrum cells were grown from frozen stocks as described above. S. aureus JE2 was grown overnight on BBL Columbia CNA agar with 5% sheep blood. Bacterial cells were harvested with sterile cotton swabs and resuspended in sterile 1× PBS to a minimal OD_600_ of 0.3 for D. pigrum and 0.1 for S. aureus. Next, 5-μl drops were individually inoculated on BHI agar medium and incubated for 2 days for D. pigrum and 1 day for S. aureus. Then, 5-μl drops of resuspended bacteria to be screened for inhibition were inoculated at different distances from the pregrown bacteria. Inhibition was assessed daily and photographically documented.

### Measurement of l-lactic acid concentration (Fig. 4A).

S. pneumoniae, S. aureus, C. pseudodiphtheriticum, and D. pigrum cells were grown from frozen stocks as described above. Cells were then harvested with a sterile cotton swab, resuspended to an OD_600_ of 0.50 in 1× PBS, and inoculated at 1:25 into BHI broth for overnight growth with gently shaking (∼50 to ∼60 rpm) at 37°C under atmospheric conditions. For mixed subcultures, i.e., C. pseudodiphtheriticum and D. pigrum, a 1:1 mixture was inoculated at 1:25 into fresh BHI broth. The overnight culture was then inoculated at 1:25 into fresh BHI broth and grown for 24 h at 37°C prior to measurement of the lactic acid concentration (mmol/liter) using a d-lactic acid/l-lactic acid kit (catalog no. 11112821035, R-Biopharm AG) per the instructions of the manufacturer and BHI as a negative control.

### Growth of S. aureus and S. pneumoniae in D. pigrum cell-free conditioned liquid medium (CFCM in Fig. 4B and C).

After growth in BHI, as described for l-lactic acid measurement, the D. pigrum KPL1914 cells were removed with a 0.22-μm-pore-size sterile filter, yielding cell-free conditioned medium (CFCM). Each of S. aureus strains Newman and JE2 and S. pneumoniae strains TIGR4, DBL5, 603, and WU2 was grown on BBL Columbia CNA agar with 5% sheep blood for 1 day, harvested with a sterile cotton swab, resuspended to an OD_600_ of 0.30 in 1× PBS, inoculated at 1:100 into both D. pigrum CFCM and BHI broth, and grown for 19 to 20 h at 37°C in shaking (S. aureus; 50 rpm) or static (S. pneumoniae) culture under atmospheric conditions. Growth yield was quantified as OD_600_ absorbance.

### Growth of S. aureus and S. pneumoniae in BHI broth supplemented with l-lactic acid (see lactic acid data in Fig. 4B and C).

Strains of S. aureus and S. pneumoniae were grown and harvested as described above for inoculation. BHI broth, supplemented with l-lactic acid (CAS no. 79-33-4; Fisher BioReagents) at various concentrations from 11 mM to 55 mM, was sterilized through a 0.22-μm-pore-size filter. After inoculation of each strain separately into BHI broth with l-lactic acid, cultures were grown as described above for growth in CFCM. Growth yield was quantified as OD_600_ absorbance.

### Growth assay for S. pneumoniae on BHI agar medium conditioned by monoculture versus coculture of D. pigrum and/or C. pseudodiphtheriticum (Fig. 5; see also Fig. S2 in the supplemental material).

D. pigrum and C. pseudodiphtheriticum strains were grown from freezer stocks as described above. Cells were harvested with sterile cotton swabs and resuspended in sterile PBS to an OD_600_ of 0.5. We then spotted 100 μl of 1:1 mixed resuspension on a polycarbonate membrane (see above) on BHI agar medium containing 400 U/ml bovine liver catalase. After 2 days of growth, the polycarbonate membrane with D. pigrum and/or C. pseudodiphtheriticum was removed from each plate, leaving CFCAM. S. pneumoniae 603 (104) was grown overnight on BBL Columbia CNA agar with 5% sheep blood as described above, and, using a sterile cotton swab, a lawn was streaked onto the CFCAM and allowed to grow for 24 h. Growth/inhibition was assessed daily and photographically recorded. Imaging was difficult due to the transparency of S. pneumoniae lawns.

### Statistical analyses.

R version 3.6.2 was used for statistical analysis and data visualization. The Wilcoxon rank sum test (equivalent to the Mann-Whitney test) was performed using wilcox.test() with paired = FALSE, alternative = “two.sided”.

### Ethics approval and consent to participate.

We isolated D. pigrum KPL1914 and C. pseudodiphtheriticum KPL1989 from the nostril of an adult as part of a protocol to study the bacterial microbiota of the nostrils of healthy adults that was initially approved by the Harvard Medical School Committee on Human Studies (105) and subsequently approved by the Forsyth Institute Institutional Review Board.

### Data availability.

We declare that all data that support the findings of this study are available within the paper (and its supplemental material); from publicly available repositories, i.e., GenBank (BioProject accession numbers PRJNA379818 and PRJNA379966); or from the corresponding authors upon reasonable request. All computer code for published tools used in this work is referenced in Materials and Methods; custom-made code (i.e., loop code) is described in Materials and Methods. Further details are available from the corresponding authors on reasonable request.
