# Supplementary figures and images for: Dolosigranulum pigrum Cooperation and Competition in Human Nasal Microbiota
Source: mSphere. 2020 Sep 9;5(5):e00852-20. doi: 10.1128/mSphere.00852-20 (PMC7485692; doi:10.1128/mSphere.00852-20)

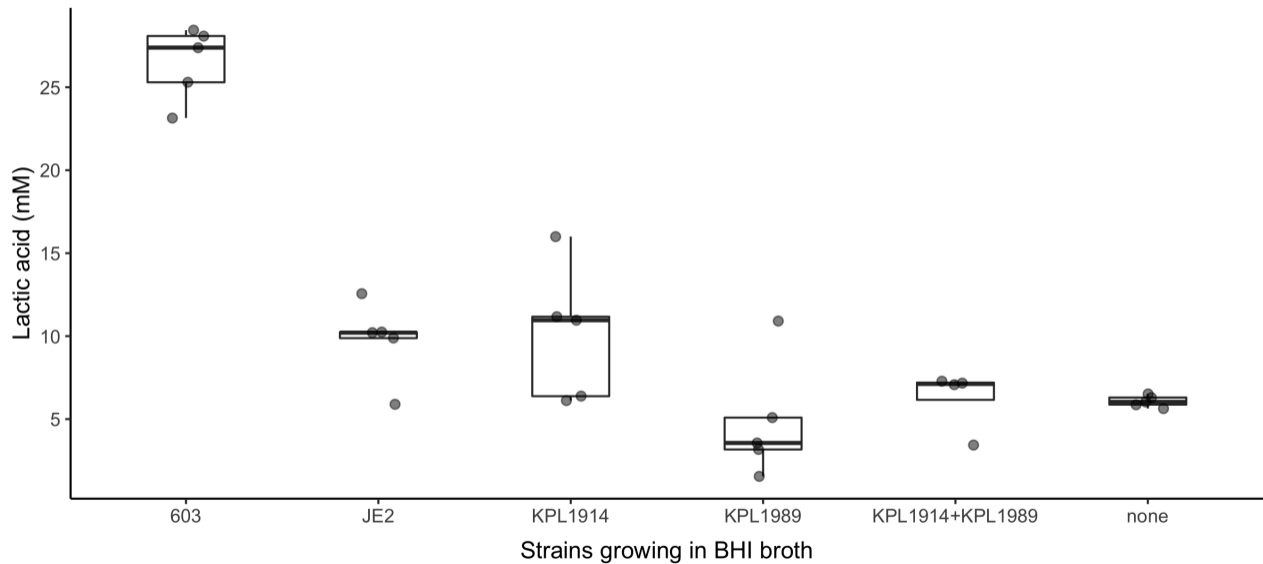

Supplement: FIG S1 [file mSphere.00852-20-sf001.pdf]

A

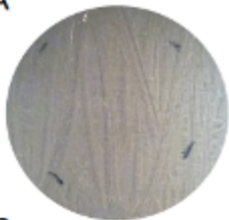

B

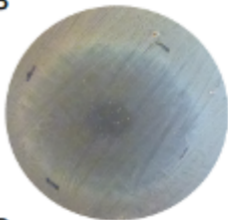

C

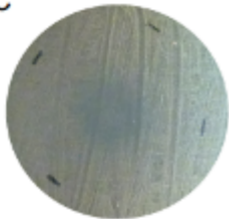

D

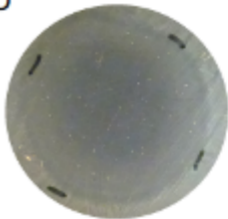

Supplement: FIG S2 [file mSphere.00852-20-sf002.pdf]
