# Supplementary material for: Dolosigranulum pigrum Cooperation and Competition in Human Nasal Microbiota
Source: mSphere. 2020 Sep 9;5(5):e00852-20. doi: 10.1128/mSphere.00852-20 (PMC7485692; doi:10.1128/mSphere.00852-20)
Supplement: TEXT S1 [file mSphere.00852-20-s0001.pdf]

Supplemental File S1. Supplemental Genomic Structure Analysis

Results: Supplemental File S1

The core genome of *D. pigrum* shows a high degree of sequence conservation. From the nucleotide sequences of the 11 *D. pigrum* strains (**Tables A** and **B**), an estimate of the upper bound of a conservative core genome for *D. pigrum* is 1200 coding sequences (CDS) based on the overlap of three ortholog prediction algorithms (**Figure A**). Two of these algorithms together estimated a lower bound of 1513 CDS for the *D. pigrum* accessory genome within a pangenome of 2729 CDS (based on an estimated core of 1216 CDS) (**Figure B** and **Table C**). Similarity matrices of the core genes and proteins from the 11 *D. pigrum* strains revealed a high degree of nucleotide (≥ 97%) and amino acid (≥ 96.8%) sequence conservation (**Figure C. i** and **C. ii**, respectively).

To gain insight into the chromosomal structure of *D. pigrum*, we closed the genomes of two strains, CDC 4709-98 and KPL1914 using SMRT sequencing. MAUVE alignment revealed large blocks of synteny between these two closed genomes (**Figure D**). Synteny analysis of the bidirectional best-hits core proteins from the RAST annotation revealed 1143 syntenic clusters (of 1206 total BDBH clusters). Ring plots using the closed genome of CDC 4709-98 as the reference (**Figure E. i**), because it is 5% (97 kb) larger than that of KPL1914 (**Figure E. ii**), showed discrete areas of sequence absence in KPL1914 (**Figure E. iii**) along with large areas of conservation. Similar areas of absence were visible when the other 9 strains were also compared to CDC 4709-98 (**Figure E. iv**).

A core-genome phylogeny reveals relationships between the 11 *D. pigrum* strains. In 16S rRNA gene-based phylogenies *D. pigrum* clades with other lactic acid bacteria and shares the closest node with *Alloiococcus otitis* (previously designated *A. otitidis*); both

species are within the family *Carnobacteriaceae* (order *Lactobacillales*) (1). Genomic content also identified *A. otitis* as its closest genome-sequenced neighbor by RAST. Using *A. otitis* ATCC 51267 as an outgroup, a core-genome, maximum likelihood-based phylogeny revealed a monophyletic *D. pigrum* clade (**Figure F**). Although the 11 strains were isolated from different individuals in different geographic regions, mostly in the late 1990s (**Table B**), there was a relatively small evolutionary distance. We next analyzed the predicted functions encoded in these *D. pigrum* genomes.

Methods: Supplemental File S1

Selection of strains and preparation of DNA for whole genome sequencing. *D. pigrum* KPL1914 was isolated from the nostril of a healthy adult. In addition, we selected 9 of 27 *D. pigrum* strains from a CDC collection (2) using an *rpoB*-based typing system with a preference for strains isolated from the nasal passages and/or from children (**Table A**). Primers Strepto F MOD (AAACTTGGACCAGAAGAAAT) and R MOD (TGTAGCTTATCATCAACCATGTG) were generated *in silico* by mapping primers Strepto F and R (3) to the *rpoB* sequence of *D. pigrum* ATCC 51524 (genome obtained from NCBI; RefSeq: NZ_AGEF00000000.1) with BLAST (4) and manually correcting misalignments in SnapGene viewer 2.8.2 (GSL Biotech, Chicago, IL). PCR were performed using extracted genomic DNA of *D. pigrum*. PCR conditions were as follows: initial denaturation 95°C for 2 minutes, then 30 cycles of denaturation for 30 seconds at 98°C, annealing at 50°C for 30 seconds, elongation 72°C for minutes and a final extension step at 72°C for 10 minutes. PCR products were cleaned using QIAquick PCR purification kit (Qiagen, Germantown, MD) and sequence determined by Sanger sequencing (Macrogen USA, Boston, MA, USA). In the genomic analysis, we also included the

publicly available genome for *D. pigrum* ATCC 51524, which was sequenced by the BROAD institute as part of the HMP (RefSeq NZ_AGEF00000000.1).

D. pigrum strains were grown atop membranes for 48 hrs as described above. Cells were harvested with a sterile tip, resuspended in 50 µl of sterile PBS and frozen at -80°C. Genomic DNA was extracted using the Epicentre MasterPure nucleic acid extraction kit (Epicentre, Madison, WI) per the manufacturer's instructions. We assessed DNA purity using a Nanodrop spectrophotometer (Nanodrop, Wilmington, DE), concentration using Qubit fluorometer (Invitrogen, Carlsbad, CA) and fragment size/quality via agarose gel electrophoresis.

Whole genome sequencing, read assembly, and annotation (Table B). Genomic DNA was sequenced at the Yale Center for Genome Analysis (YCGA), New Haven, CT, on an Illumina MiSeq platform using paired-end (2 x 250 bp) technology, assembled using de Bruijn graph algorithms with Velvet (5) with a kmer size of 139 bp and annotated with RAST with FIGfam release 70 (6) and Prokka (7). In addition, *D. pigrum* strains KPL1914 and CDC 4709-98 (2) were sequenced on a PacBio RS II (Pacific Biosystems, Menlo Park, CA) and sequences were assembled using HGAP version 3.0 (8). We used an iterative procedure to error correct the PacBio genomes, which involved mapping Illumina reads to the PacBio genomes until there were no differences detected between the Illumina reads and the PacBio assembly (9). To estimate the degree of assembly errors and missing content that might contribute to the variation in gene content, we compared the Illumina assembly of KPL1914 with the Illumina-corrected PacBio assembly of KPL1914 to estimate the possible divergence (10). Within Illumina assemblies, we identified 139 (1566 vs. 1705) predicted coding sequences as determined by RAST

annotation absent in the assembly received by PacBio sequencing. Genomes were deposited at NCBI (GenBank: NAJJ00000000, NAQW00000000, NAQX00000000, NAQV00000000, NAQU00000000, NAQT00000000, NAQS00000000, NAQR00000000, NAQQ00000000 and NAQP00000000 in BioProjects PRJNA379818 and PRJNA379966).

Identification of the *D. pigrum* core, shell and cloud genome based on Illumina-sequenced genomes from 11 strains (Figures A and B and Table C). Core proteins from RAST-annotated GenBank-files were determined using the intersection of bidirectional best-hits (BDBH), cluster of orthologous (COG) triangles and Markov Cluster Algorithm (OrthoMCL) clustering algorithms using GET_HOMOLOGUES package version 02012019 on Ubuntu-Linux (11). Clustering for each of the three algorithms was performed with ./get_homologues.pl (with default parameters) and the intersection of the three was calculated with ./compare_clusters.pl excluding proteins with more than one copy in an input species, as single-copy proteins are safer orthologues, (i.e., flag -t 11). GenBank files derived from RAST annotation (see above) were renamed with KPL strain names except for strain ATCC51524. We determined the cloud, shell and core genome of each of the 11 sequenced *D. pigrum* strains using the parse_pangenome_matrix.pl script (./parse_pangenome_matrix.pl -m sample_intersection/pangenome_matrix_t0.tab -s) of the GET_HOMOLOGUES package version 02012019 (11). Definition of cloud, shell and core genome were based on (12). In brief, cloud is defined as genes only present in 1 or 2 genomes (cut-off is defined as the class next to the most populated non-core cluster class). The core genome is composed of clusters present in all 11 strains, soft core contains clusters present in 10 genomes and shell includes clusters present in 3 to 9

genomes. Synteny analysis (**Figure D**) on BDBH core (with flag -t 11) was performed using the compare_clusters script (-s) and synteny visualization was done in MAUVE using standard settings (13) after the KPL1914 genome was reverse complemented and both genomes had the origin set at the beginning of *dnaA*.

Phylogenetic reconstruction, sequence and protein similarities. A monophyletic (clade) core genome phylogenic tree was constructed by including *A. otitis* (closest neighbor based on the Living Tree Project (1)) an outgroup (**Figure F. i**). A phylogenic tree without an outgroup was also constructed similarly (**Figure F. ii**). *A. otitis* ATCC 51267 contigs were downloaded from NCBI (NZ_AGXA00000000.1) and annotated using RAST (see above). Predicted core proteins common to *A. otitis* and *D. pigrum* genomes were identified as described above using GET_HOMOLOGUES package version 02012019. Alignments were done using a loop with Clustal Omega V. 1.2.4 ($ for filename in *.faa; do clustalo -i "$filename" -o clustalo_out/${filename%coral} -v; done) and resulting alignments were concatenated using catfasta2phyml perl script (https://github.com/nylander/catfasta2phyml) $./catfasta2phyml.pl *.faa --verbose > outv.phy. PhyML 3.0 (14) with smart model selection (15) using Akaike information criterion was used for phylogenetic analysis (maximum-likelihood) with 100 regular bootstrap replicates and FigTree (http://tree.bio.ed.ac.uk/software/figtree/) for tree visualization.

BLAST Ring Image Generator (BRIG) was used for visualization of the other sequenced genomes compared to the closed CDC 4709-98 genome (**Figure E**) (16). Average amino acid and nucleic acid identity (**Figure C**) was calculated using GET_HOMOLOGUES package version 30062017 (11). In brief, a pangenome matrix was generated using the

OMCL algorithm (./get_homologues.pl -d dpig_folder -t 0 -M (OMCL)) for homologues identification. Both, ANI and AAI were calculated with all available clusters (-t 0). Commands used: Generation of an AA identity matrix: $ ./get_homologues.pl -d “gbk-files” -A -t 0 -M and CDS identity matrix with the command $./get_homologues.pl -d “gbk files” -a 'CDS' -A -t 0 -M.

Table A. Characteristics of *D. pigrum* strains and Illumina genomes in this study.

Strain name / (internal strain #)	GB Assembly (BioProject)	Strain source	Geography / body site / age	Median fold coverage	CDS[+]	RNAs[+]	GC (%)[+]	Size (bp)
KPL1914 (KPL1914)	GCA_003263915.2 [#] (PRJNA379818)	This study	MA / nostril / adult	83	1566	72	40	1,726,398
CDC 39-95 (KPL1922)	GCA_003264145.1 (PRJNA379966)	CDC (2)	Canada / sinus / 3 years	62	1666	80	39.7	1,859,258
CDC 2949-98 (KPL1930)	GCA_003264135.1 (PRJNA379966)	CDC (2)	AZ / nasopharyngeal / NA	60	1644	73	39.6	1,886,398
CDC 4294-98 (KPL1931)	GCA_003264085.1 (PRJNA379966)	CDC (2)	SC / blood / 2 months	73	1841	78	39.5	2,014,679
CDC 4420-98 (KPL1932)	GCA_003264065.1 (PRJNA379966)	CDC (2)	TN / blood / 11 years	63	1745	73	39.7	1,934,436
CDC 4545-98 (KPL1933)	GCA_003264045.1 (PRJNA379966)	CDC (2)	AZ / nasopharyngeal / NA	128	1680	61	39.6	1,861,299
CDC 4709-98 (KPL1934)	GCA_003264015.2 [#] (PRJNA379966)	CDC (2)	GA / eye / 2 months	81	1686	80	39.6	1,912,682
CDC 4199-99 (KPL1937)	GCA_003264005.1 (PRJNA379966)	CDC (2)	GA / blood / 1.8 years	107	1746	85	39.6	1,976,602
CDC 4791-99 (KPL1938)	GCA_003263975.1 (PRJNA379966)	CDC (2)	AZ / nasopharyngeal / NA	61	1651	80	39.6	1,873,869
CDC 4792-99 (KPL1939)	GCA_003263965.1 (PRJNA379966)	CDC (2)	AZ / nasopharyngeal / NA	92	1704	80	39.4	1,893,917
SS-1342 [NCFB2967] [ATCC 51524]	GCA_000245815.1 *	BROAD/HMP R91/1468 (17)	England / spinal cord (autopsy)	200	1651	31	39.6	1,846,028

[#] version .1 is the Illumina assembly and version .2 is the Illumina-corrected PacBio assembly
[+] as determined by RAST annotation (see text)
* sequenced by the BROAD institute for the Human Microbiota Project

Table B. Assembly characteristics of *D. pigrum* Illumina genomes in this study.

CDC / internal strain #		Nodes [N]	N50 [bp]	Max [bp]	Total [bp]	Reads [N]
	KPL1914	107	87,900	210,575	1,726,398	1,640,818
39-95	KPL1922	138	153,015	273,357	1,859,258	1,819,350
2949-98	KPL1930	179	127,744	456,484	1,886,398	1,699,318
4294-98	KPL1931	139	88,498	198,469	2,014,679	2,357,776
4420-98	KPL1932	134	209,743	328,871	1,934,436	2,098,746
4545-98	KPL1933	50	283,724	492,087	1,861,299	2,738,030
4709-98	KPL1934	142	110,284	268,980	1,912,682	2,198,156
4199-99	KPL1937	109	128,019	379,812	1,976,602	2,528,652
4791-99	KPL1938	129	132,767	316,186	1,873,869	1,831,854
4792-99	KPL1939	86	253,067	460,850	1,893,917	2,039,094

Figure A. The conservative core genome of 11 *D. pigrum* strains encodes 1200 orthologs. A Venn diagram generated using the bidirectional best-hits (BDBH), cluster of orthologous groups (COG) triangle, and OrthoMCL (OMCL) algorithms identified predicted protein orthologs (RAST annotation) shared between the 11 *D. pigrum* genomes (GET_HOMOLOGUES package version 02012019) (11). Flag -t 11 was used to only include clusters containing single-copy orthologs from all input species since these are likely the most reliable ortholog predictions.

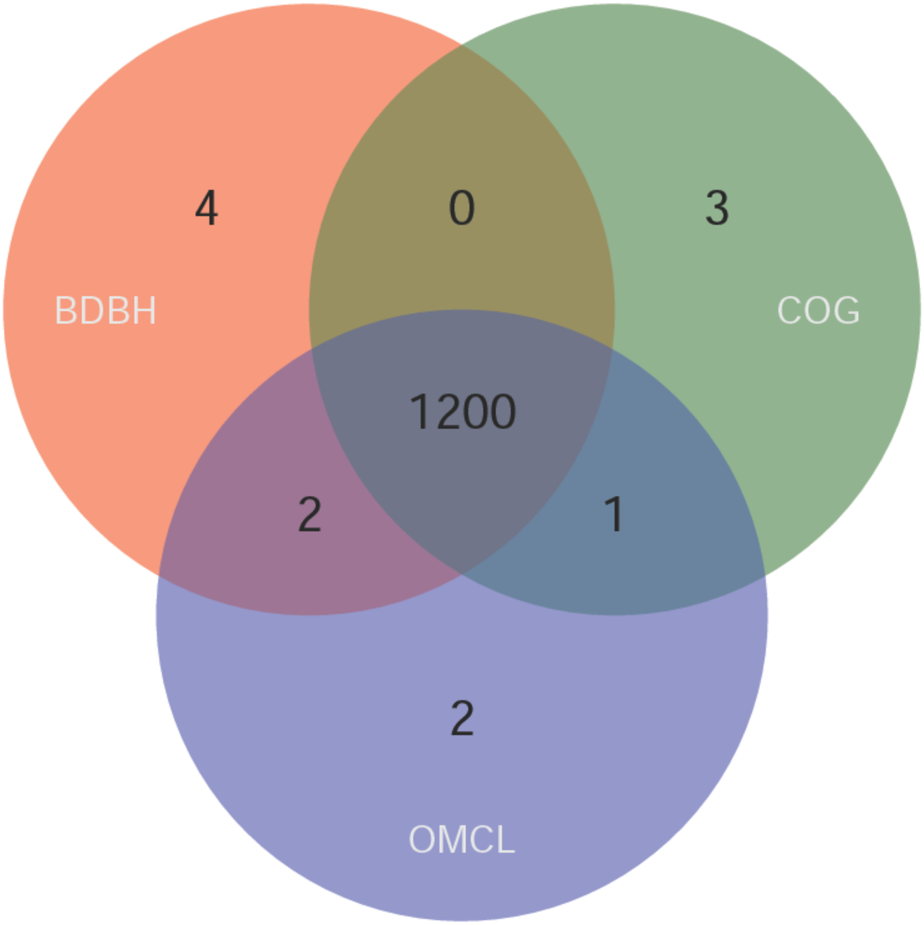

Figure B. Core, shell and cloud pangenome of 11 *Dolosigranulum pigrum* strains. The pangenome of the 11 *D. pigrum* strains includes an estimated core of 1216, soft core of 116, shell of 373 and cloud of 1024 genes (CDS), as determined by the parse_pangenome_matrix.pl script (using the OMCL / COG intersection) of the GET_HOMOLOGUES package version 02012019 (11). The core genome is composed of genes that are present in all strains and soft core contains clusters present in 10 genomes but not the core as defined in (12). Cloud is defined as genes only present in a few genomes (cut-off is defined as the class next to the most populated non-core cluster class). Shell genes are the remaining genes and displayed sorted to the number of genomes in which these are present (*n* = 3-9).

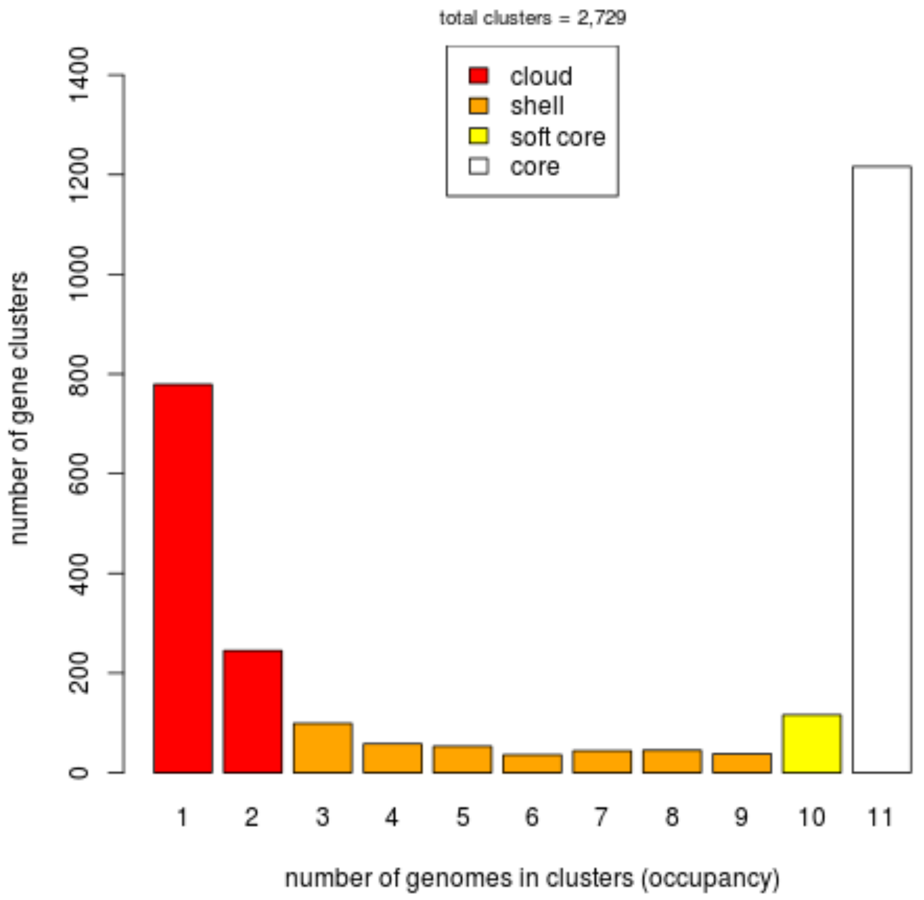

Table C. Distribution by strain of genes in the core, soft core, shell and cloud *D. pigrum* genome.*

CDC or other strain #	Internal reference	Cloud [N]	Shell [N]	Soft core [N]	Core [N]
KPL1914	KPL1914	79	135	89	1216
39-95	KPL1922	81	189	109	1216
2949-98	KPL1930	88	167	113	1216
4294-98	KPL1931	243	183	110	1216
4420-98	KPL1932	134	205	114	1216
4545-98	KPL1933	102	202	96	1216
4709-98	KPL1934	105	185	111	1216
4199-99	KPL1937	186	174	94	1216
4791-99	KPL1938	73	192	108	1216
4792-99	KPL1939	110	194	108	1216
ATCC51524		68	194	108	1216

* These results were determined as described for Figure S3.

Figure C. The (i) average nucleotide identities (ANI) and (ii) amino acid identities (AAI) show a high degree of conservation across all 11 *D. pigrum* genomes. Average identity matrices of clustered coding sequences were calculated using GET_HOMOLOGUES with the OrthoMCL algorithm. Both ANI and AAI were calculated with all available clusters (-t 0).

i

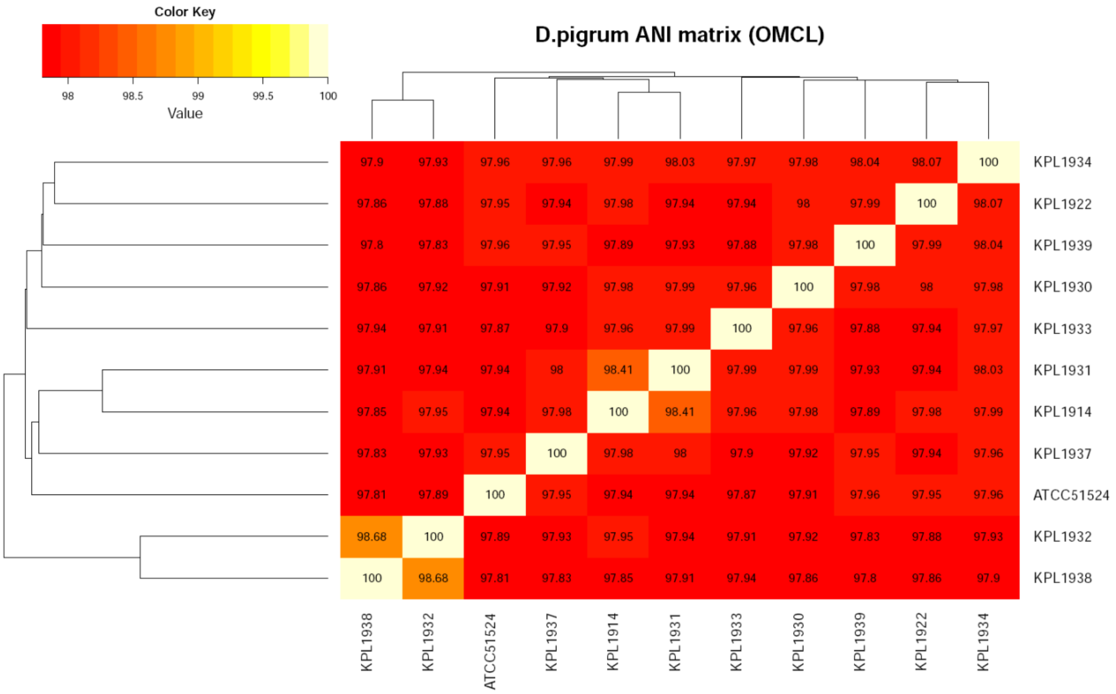

ii

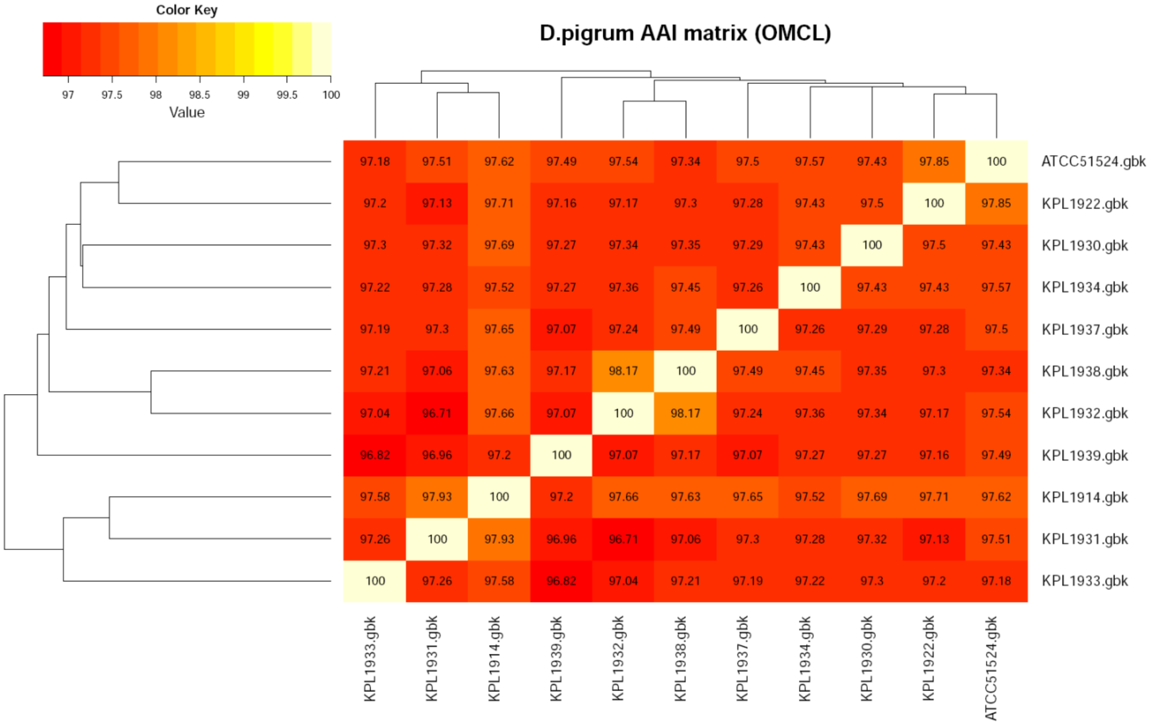

Color Key
97 97.5 98 98.5 99 99.5 100
Value
D.pigrum AAI matrix (OMCL)
97.18 97.51 97.62 97.49 97.54 97.34 97.5 97.57 97.43 97.85 100 ATCC51524.gbk
97.2 97.13 97.71 97.16 97.17 97.3 97.28 97.43 97.5 100 97.85 KPL1922.gbk
97.3 97.32 97.69 97.27 97.34 97.35 97.29 97.43 100 97.5 97.43 KPL1930.gbk
97.22 97.28 97.52 97.27 97.36 97.45 97.26 100 97.43 97.43 97.57 KPL1934.gbk
97.19 97.3 97.65 97.07 97.24 97.49 100 97.26 97.29 97.28 97.5 KPL1937.gbk
97.21 97.06 97.63 97.17 98.17 100 97.49 97.45 97.35 97.3 97.34 KPL1938.gbk
97.04 96.71 97.66 97.07 100 98.17 97.24 97.36 97.34 97.17 97.54 KPL1932.gbk
96.82 96.96 97.2 100 97.07 97.17 97.07 97.27 97.27 97.16 97.49 KPL1939.gbk
97.58 97.93 100 97.2 97.66 97.63 97.65 97.52 97.69 97.71 97.62 KPL1914.gbk
97.26 100 97.93 96.96 96.71 97.06 97.3 97.28 97.32 97.13 97.51 KPL1931.gbk
100 97.26 97.58 96.82 97.04 97.21 97.19 97.22 97.3 97.2 97.18 KPL1933.gbk
KPL1933.gbk KPL1931.gbk KPL1914.gbk KPL1939.gbk KPL1932.gbk KPL1938.gbk KPL1937.gbk KPL1934.gbk KPL1930.gbk KPL1922.gbk ATCC51524.gbk

Figure D. Comparative analysis of two *D. pigrum* genomes reveals a high degree of synteny. Multiple genome alignment for synteny analysis of the two closed *D. pigrum* genomes CDC 4709-98 and KPL1914 was performed using MAUVE (13). Locally collinear blocks (color coded) and similarity profiles are presented, and genome boundaries are indicated (red bar after non-aligned sequences, i.e., sequences that are unique for the corresponding genome).

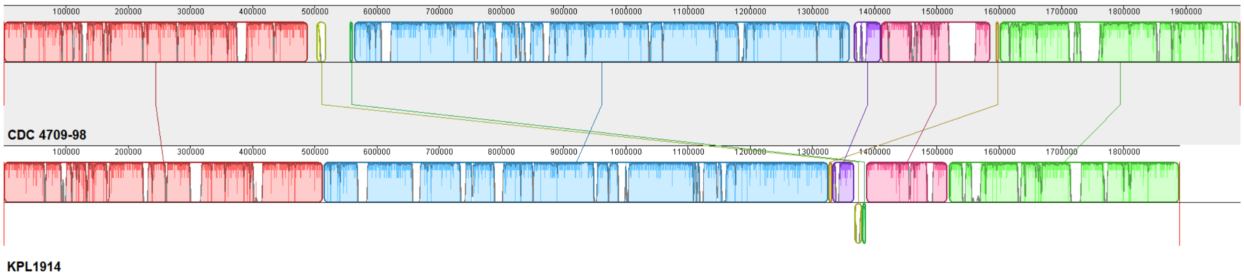

Figure E. *D. pigrum* genomes BLAST ring comparisons. BLAST Ring Image Generator (BRIG) version 0.95 (16) was used for visualization of the sequenced genomes with the closed genome of CDC 4709-98 as a reference. (**i**) Closed genome of CDC 4709-98 with GC plots (**ii**) Closed genome of KPL 1914 with GC plots (**iii**) BLASTN based comparison of the closed genomes of CDC 4709-98 and KPL 1914 (**iv**) BLASTN based comparison of CDC 4709-98 and the remaining 10 Illumina contig-based genomes as well as ATCC 51524.

i

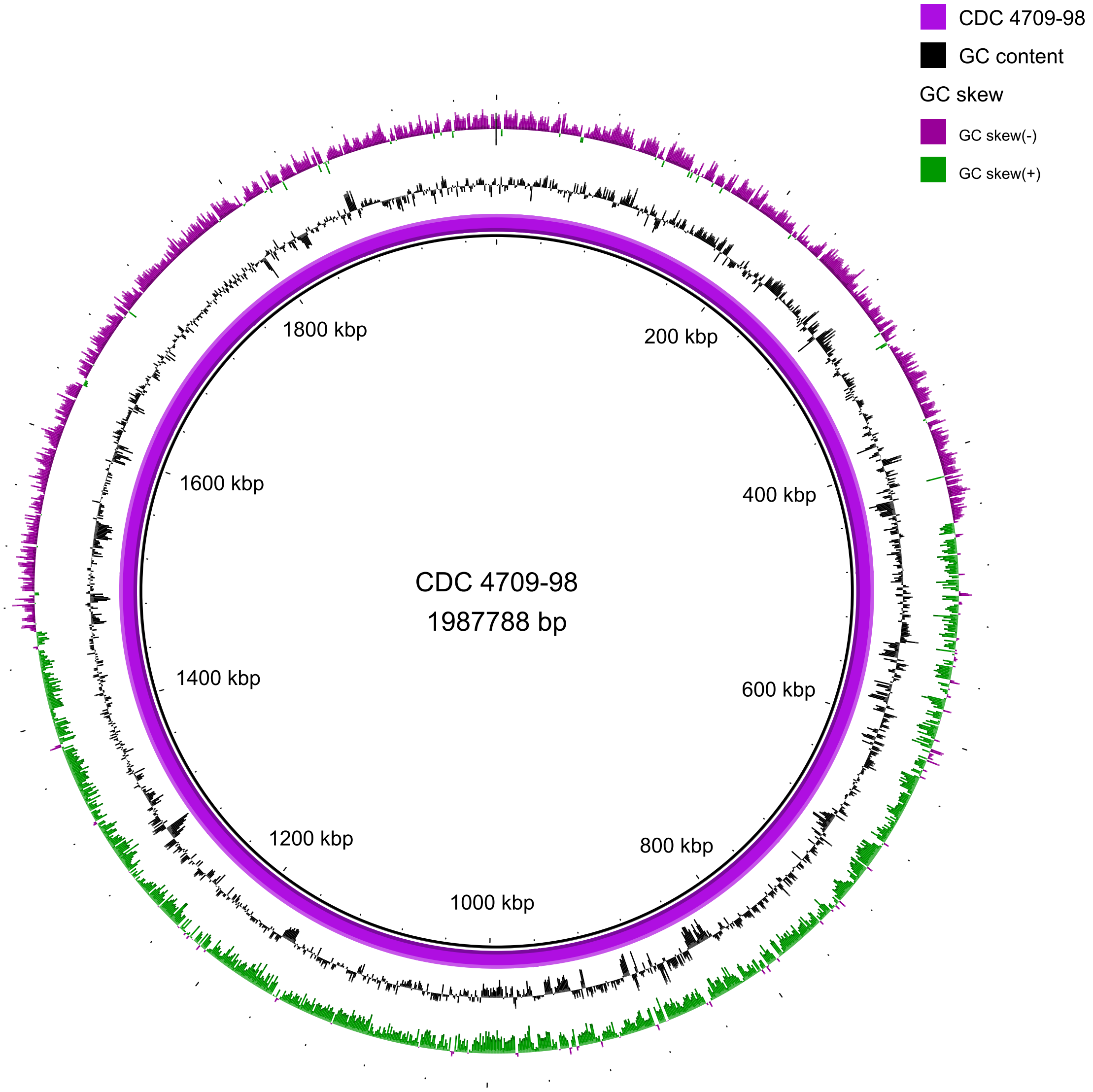

ii

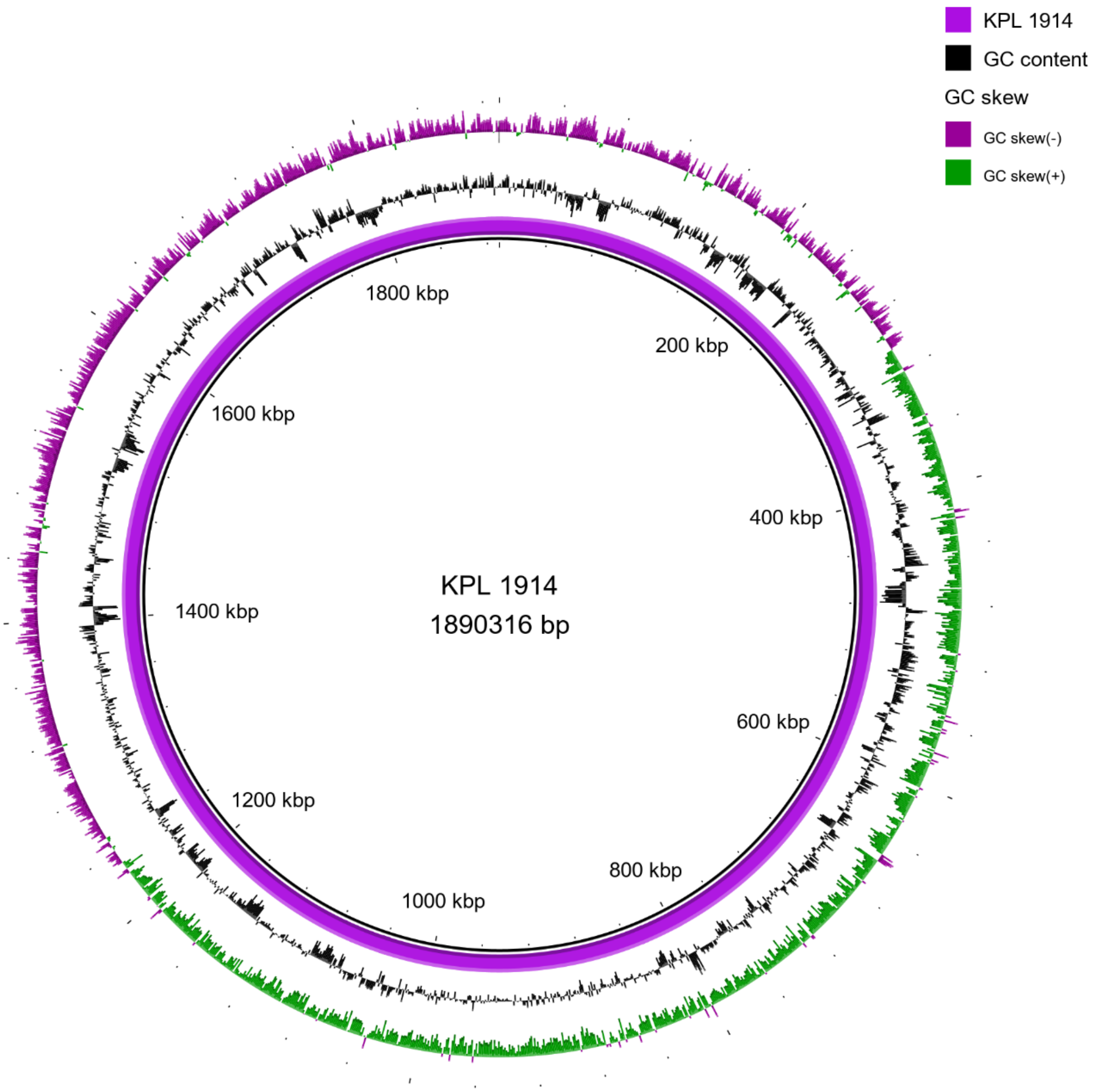
KPL 1914
GC content
GC skew
GC skew(-)
GC skew(+)
KPL 1914
1890316 bp
200 kbp
400 kbp
600 kbp
800 kbp
1000 kbp
1200 kbp
1400 kbp
1600 kbp
1800 kbp

iii

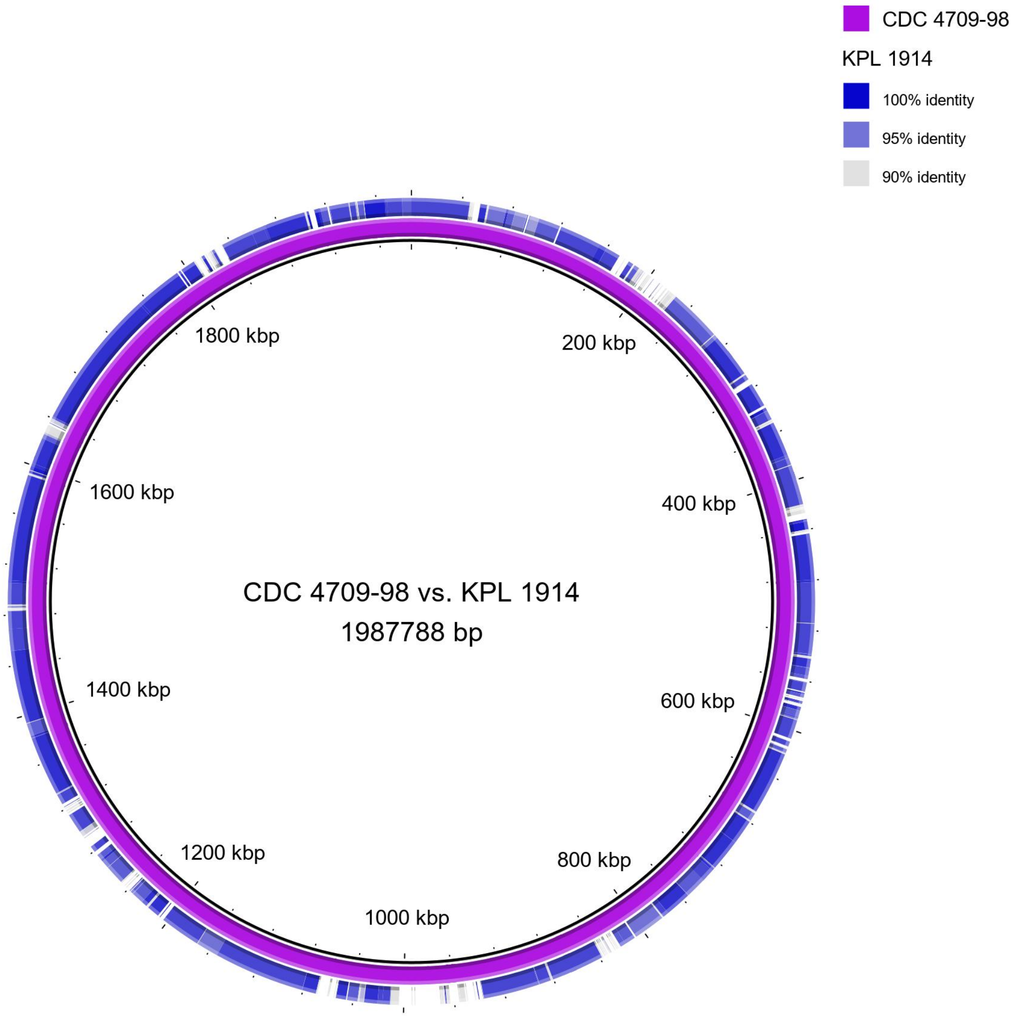
CDC 4709-98
KPL 1914
100% identity
95% identity
90% identity
CDC 4709-98 vs. KPL 1914
1987788 bp
200 kbp
400 kbp
600 kbp
800 kbp
1000 kbp
1200 kbp
1400 kbp
1600 kbp
1800 kbp

iv

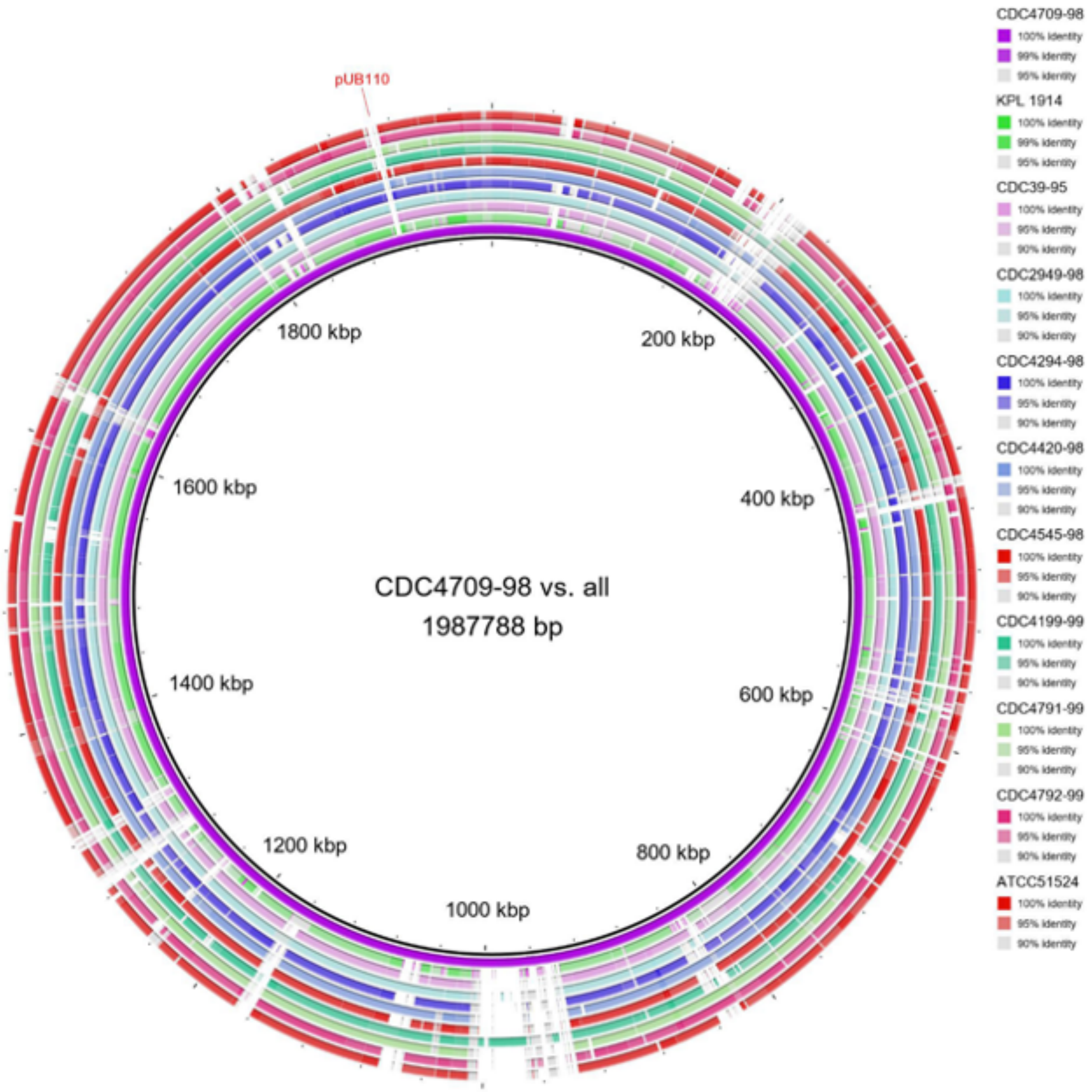
pUB110
1800 kbp
200 kbp
1600 kbp
400 kbp
CDC4709-98 vs. all
1987788 bp
1400 kbp
600 kbp
1200 kbp
800 kbp
1000 kbp
CDC4709-98
100% identity
99% identity
95% identity
KPL 1914
100% identity
99% identity
95% identity
CDC39-95
100% identity
95% identity
90% identity
CDC2949-98
100% identity
95% identity
90% identity
CDC4294-98
100% identity
95% identity
90% identity
CDC4420-98
100% identity
95% identity
90% identity
CDC4545-98
100% identity
95% identity
90% identity
CDC4199-99
100% identity
95% identity
90% identity
CDC4791-99
100% identity
95% identity
90% identity
CDC4792-99
100% identity
95% identity
90% identity
ATCC51524
100% identity
95% identity
90% identity

Figure F. Phylogenetic relationship between the 11 *D. pigrum* strains. *D. pigrum* phylogenetic trees were constructed by using concatenated alignments of the intersection of predicted core genes (BDBH, COG, and OMCL, see above). Amino acids were aligned using Clustal Omega V.1.2.4 (18) and a maximum likelihood phylogenetic tree was generated using the LG model of amino-acid replacement matrix (19) as selected by smart model selection with Akaike information criterion (15) and 100 bootstrap replicates for branch support with PhyML (phylogenetic maximal likelihood) V. 3.0 (14) and visualized using FigTree V.1.4.4. A BIONJ distance-based tree was used as a starting tree to be redefined by the maximum likelihood algorithm. Bootstrap support values from 100 replicates are indicated above the branches. (**i**) Core genome amino acid tree of the 11 *D. pigrum* genomes. (**ii**) Core genome amino acid tree with *A. otitis* ATCC 51267 as an outgroup. Core genome size decreased to 866 clusters when including *A. otitis*.

i

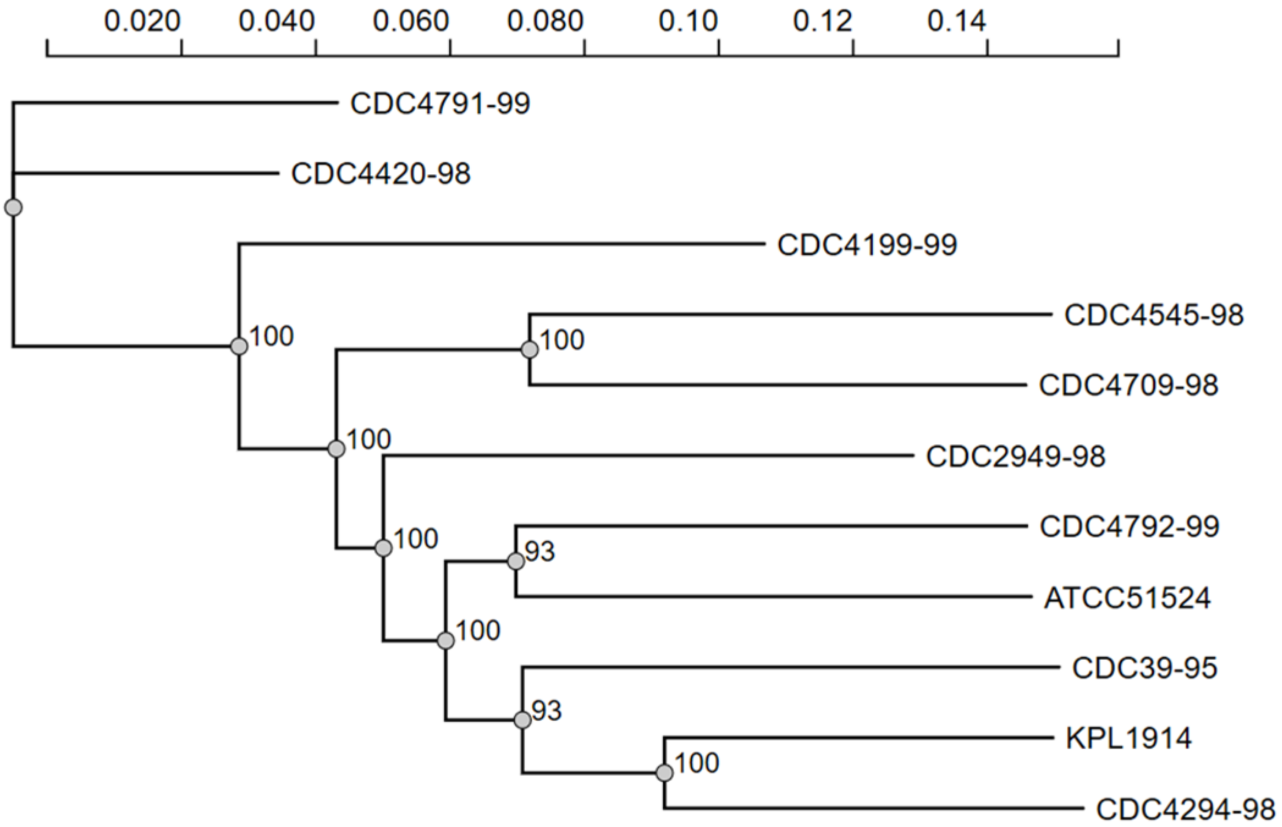
0.020
0.040
0.060
0.080
0.10
0.12
0.14
CDC4791-99
CDC4420-98
CDC4199-99
100
CDC4545-98
100
CDC4709-98
100
CDC2949-98
100
CDC4792-99
93
ATCC51524
100
CDC39-95
93
KPL1914
100
CDC4294-98

ii

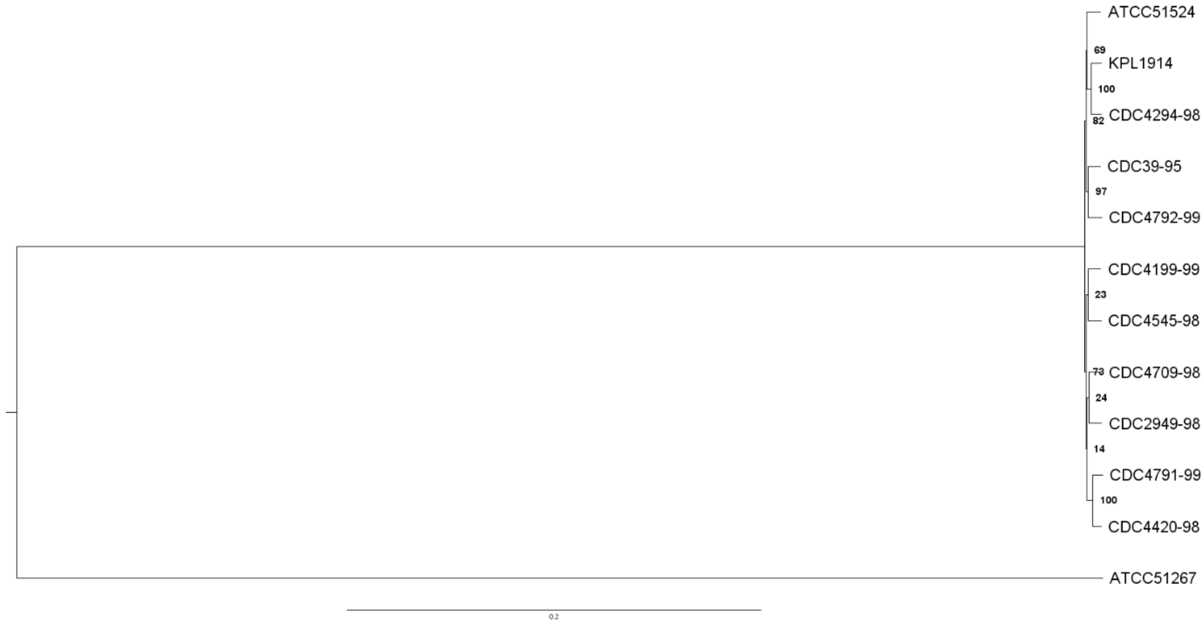
ATCC51524
69
KPL1914
100
82
CDC4294-98
CDC39-95
97
CDC4792-99
CDC4199-99
23
CDC4545-98
73
CDC4709-98
24
CDC2949-98
14
CDC4791-99
100
CDC4420-98
ATCC51267

References: Supplemental File S1

1. Yarza P, Richter M, Peplies J, Euzeby J, Amann R, Schleifer KH, Ludwig W, Glockner FO, Rossello-Mora R. 2008. The All-Species Living Tree project: a 16S rRNA-based phylogenetic tree of all sequenced type strains. Syst Appl Microbiol 31:241-50.

2. Laclaire L, Facklam R. 2000. Antimicrobial susceptibility and clinical sources of *Dolosigranulum pigrum* cultures. Antimicrob Agents Chemother 44:2001-3.

3. Drancourt M, Roux V, Fournier PE, Raoult D. 2004. rpoB gene sequence-based identification of aerobic Gram-positive cocci of the genera *Streptococcus*, *Enterococcus*, *Gemella*, *Abiotrophia*, and *Granulicatella*. J Clin Microbiol 42:497-504.

4. Altschul SF, Gish W, Miller W, Myers EW, Lipman DJ. 1990. Basic local alignment search tool. J Mol Biol 215:403-10.

5. Zerbino DR, Birney E. 2008. Velvet: algorithms for *de novo* short read assembly using de Bruijn graphs. Genome Res 18:821-9.

6. Aziz RK, Bartels D, Best AA, DeJongh M, Disz T, Edwards RA, Formsma K, Gerdes S, Glass EM, Kubal M, Meyer F, Olsen GJ, Olson R, Osterman AL, Overbeek RA, McNeil LK, Paarmann D, Paczian T, Parrello B, Pusch GD, Reich C, Stevens R, Vassieva O, Vonstein V, Wilke A, Zagnitko O. 2008. The RAST Server: rapid annotations using subsystems technology. BMC Genomics 9:75.

7. Seemann T. 2014. Prokka: Rapid prokaryotic genome annotation. Bioinformatics 30:2068-9.

8. Chin CS, Alexander DH, Marks P, Klammer AA, Drake J, Heiner C, Clum A, Copeland A, Huddleston J, Eichler EE, Turner SW, Korlach J. 2013. Nonhybrid, finished microbial genome assemblies from long-read SMRT sequencing data. Nat Methods 10:563-9.

9. Pettigrew MM, Ahearn CP, Gent JF, Kong Y, Gallo MC, Munro JB, D'Mello A, Sethi S, Tettelin H, Murphy TF. 2018. *Haemophilus influenzae* genome evolution during persistence in the human airways in chronic obstructive pulmonary disease. Proc Natl Acad Sci U S A 115:E3256-E3265.

10. Hilty M, Wuthrich D, Salter SJ, Engel H, Campbell S, Sa-Leao R, de Lencastre H, Hermans P, Sadowy E, Turner P, Chewapreecha C, Diggle M, Pluschke G, McGee L, Eser OK, Low DE, Smith-Vaughan H, Endimiani A, Kuffer M, Dupasquier M, Beaudoing E, Weber J, Bruggmann R, Hanage WP, Parkhill J, Hathaway LJ, Muhlemann K, Bentley SD. 2014. Global phylogenomic analysis of nonencapsulated *Streptococcus pneumoniae* reveals a deep-branching classic

lineage that is distinct from multiple sporadic lineages. Genome Biol Evol 6:3281-94.

11. Contreras-Moreira B, Vinuesa P. 2013. GET_HOMOLOGUES, a versatile software package for scalable and robust microbial pangenome analysis. Appl Environ Microbiol 79:7696-701.

12. Kaas RS, Friis C, Ussery DW, Aarestrup FM. 2012. Estimating variation within the genes and inferring the phylogeny of 186 sequenced diverse *Escherichia coli* genomes. BMC Genomics 13:577.

13. Darling AE, Mau B, Perna NT. 2010. progressiveMauve: multiple genome alignment with gene gain, loss and rearrangement. PLoS One 5:e11147.

14. Guindon S, Dufayard JF, Lefort V, Anisimova M, Hordijk W, Gascuel O. 2010. New algorithms and methods to estimate maximum-likelihood phylogenies: assessing the performance of PhyML 3.0. Syst Biol 59:307-21.

15. Lefort V, Longueville JE, Gascuel O. 2017. SMS: Smart Model Selection in PhyML. Mol Biol Evol 34:2422-2424.

16. Alikhan NF, Petty NK, Ben Zakour NL, Beatson SA. 2011. BLAST Ring Image Generator (BRIG): simple prokaryote genome comparisons. BMC Genomics 12:402.

17. Aguirre M, Morrison D, Cookson BD, Gay FW, Collins MD. 1993. Phenotypic and phylogenetic characterization of some *Gemella*-like organisms from human infections: description of *Dolosigranulum pigrum* gen. nov., sp. nov. J Appl Bacteriol 75:608-12.

18. Sievers F, Wilm A, Dineen D, Gibson TJ, Karplus K, Li W, Lopez R, McWilliam H, Remmert M, Soding J, Thompson JD, Higgins DG. 2011. Fast, scalable generation of high-quality protein multiple sequence alignments using Clustal Omega. Mol Syst Biol 7:539.

19. Le SQ, Gascuel O. 2008. An improved general amino acid replacement matrix. Mol Biol Evol 25:1307-20.
