# Supplementary material for: Dolosigranulum pigrum Cooperation and Competition in Human Nasal Microbiota
Source: mSphere. 2020 Sep 9;5(5):e00852-20. doi: 10.1128/mSphere.00852-20 (PMC7485692; doi:10.1128/mSphere.00852-20)
Supplement: TEXT S2 [file mSphere.00852-20-s0002.pdf]

Supplemental File S2. Supplemental Genomic Functional Predictions

Results: Supplemental File S2

I. Predicted biosynthesis, uptake and degradation by *D. pigrum* of amino acids, carbohydrates, polyamines and enzyme co-factors

Methionine auxotrophy and degradation. All 11 *D. pigrum* genomes lacked a complete known pathway for methionine biosynthesis. Methionine has been reported to be a limiting nutrient in the nasal passages of humans and its synthesis is upregulated in *S. aureus* growing in synthetic nasal medium (1). In contrast, all 11 encoded two full sets of the genes required for methionine degradation in different regions on their chromosome suggesting external dependence. Each set includes *metN* (methionine ABC transporter ATP-binding protein), *metP* (methionine ABC transporter permease protein), *metQ* (methionine ABC transporter substrate-binding protein) and *metT* (methionine transporter).

Arginine auxotrophy and degradation. All 11 *D. pigrum* strains lacked most of the genes required for synthesis of arginine from glutamate (e.g., *argB, argC, argD, argF, argG* and *argH*) suggesting likely auxotrophy. All 11 strains contained the genes of the arginine deiminase pathway (*arcA, arcB, arcC, arcD* and *arcR*) suggesting *D. pigrum* may uptake extracellular arginine.

Glutamine. Glutamine synthetase I (EC 6.3.1.2) was predicted in all 11 strains and catalyzes the reaction of L-glutamine from L-glutamate and NH_4^+ using ATP. Glutamate racemase (EC 5.1.1.3) was predicted in all 11 and catalyzes D- / L-glutamate interconversion. NAD(P)-specific glutamate dehydrogenase (EC 1.1.1.4) GdhA was

predicted in all 11 and catalyzes the formation of L-glutamate and H_2O from 2-oxoglutarate, ammonia and NADH + H^+ and vice versa.

Polyamine auxotrophy and transport. The absence of predicted genes required for *de novo* synthesis of the essential polyamines putrescine and spermidine indicates that *D. pigrum* likely relies on extracellular polyamines. Consistent with this, all strains harbor putative ABC type spermidine transporter components (SPERta, SPERtb, SPERtc, SPERtd) adjacent to each other as well as putative putrescine transporter genes (*potA*, *potB*, *potC*, and *potD*).

Biotin auxotrophy and transport. A biotin uptake system was predicted in all 11 *D. pigrum* strains (substrate-specific component BioY of biotin ECF transporter) and no biosynthesis was predicted. Biotin-protein ligase (EC 6.3.4.15) was predicted only in KPL1914 and ATCC51524.

Nicotinic acid (niacin) auxotrophy and transport. NAD and NADP are critical factors for cellular metabolism**.** However, *D. pigrum* lacked known genes for *de novo* synthesis and for salvage of nicotinate or nicotinamide. However, the presence of *niaP* predicts uptake of nicotinic acid (niacin) by all 11 strains. All strains also encoded genes needed to convert niacin or nicotinamide to NAD+ and NADP: nicotinamidase (EC 3.5.1.19), nicotinate phosphoribosyltransferase (EC 2.4.2.11), nicotinate-nucleotide adenylyltransferase (EC 2.7.7.18), NAD synthetase (EC 6.3.1.5), NAD kinase (EC 2.7.1.23) and NadR transcriptional regulator.

***D. pigrum* encodes mechanisms for acquiring essential metal cofactors from the host environment.** The nasal environment is low on essential metal ions such as iron, zinc and manganese and host metal sequestration using lactoferrin and calprotectin is an

important defense mechanism against bacterial growth. Bacteria have acquired mechanisms to escape this nutritional immunity (1, 2). We therefore searched for genes predicted to encode for siderophores and transporters for heme, manganese and zinc. All 11 *D. pigrum* genomes harbored a predicted iron compound ABC uptake transporter ATP-binding protein (hemin uptake system subsystem) and a manganese ABC-type transporter. Additionally, six of the CDC strains (4294-98, 4420-98, 4545-98, 4199-99, 4791-99, 4792-99) had predicted ferric iron ABC transporter and/or iron compound ABC uptake transporter genes.

II. Predicted carbohydrate metabolism by *D. pigrum* via homofermentation to lactate

There is no tricarboxylic acid (TCA) cycle present in *D. pigrum*. Only fumarate-hydratase (EC 4.2.1.2) and TCA associated dihydrolipoyl dehydrogenase (EC 1.8.1.4) were predicted in all *D. pigrum* genomes.

Anaerobic respiratory reductases. We did not identify butyryl-CoA-reductase (EC 1.3.8.1) or any other predicted anaerobic reductases in all *D. pigrum* strains. However, *D. pigrum* CDC 4545-98 encoded an arsenate reductase (EC 1.20.4.1).

Identification of a V-type ATPase in all 11 isolates. V-ATPases hydrolyse ATP to drive a proton pump but cannot work in reverse to synthesize ATP.

Glycolysis (Embden-Meyerhof-Parnas pathway, EMP). Enzymes present in all 11 isolates included glucokinase, glucose-6-phosphate isomerase, 6-phosphofructokinase, fructose-bisphosphate aldolase class II-1,6-bisphosphate-aldolase, triose phosphate isomerase, NAD-dependent glyceraldehyde-3-phosphate dehydrogenase,

phosphoglycerate kinase, 2,3-bisphosphoglycerate-independent phosphoglycerate mutase, enolase and pyruvate kinase. Three strains (KPL1914, CDC 39-95, CDC 4792-99) also encoded a predicted fructose-bisphosphate aldolase class I (EC 4.1.2.13), whereas all strains harbored a predicted triosephosphate isomerase.

As noted in the main text, all 11 strains encoded a predicted L-lactate-dehydrogenase (EC 1.1.1.27), which catalyzes the reduction of pyruvate to lactate regenerating NAD+ for glycolysis (GAPDH step), consistent with homofermentation to L-lactate as the primary product of glycolysis. Moreover, the absence of xylulose-5-phosphate phosphoketolase (EC 4.1.2.9) is inconsistent with (obligate) heterofermentation, since bacteria that heteroferment lack aldolase and have to shunt through the pentose phosphate or phosphoketolase pathway (3). In addition to homofermentation to lactate, some end product flexibility, which is probably produced under differing environmental/nutritional conditions, is predicted by the presence in all 11 genomes of genes needed to accomplish mixed-acid fermentation with potential production of formate, acetate and ethanol (i.e., enzymes pyruvate formate-lyase (EC 2.3.1.54), phosphate acetyltransferase (EC 2.3.1.8) and acetate kinase (EC 2.7.2.1), as well as acetaldehyde dehydrogenase (1.2.1.10) and alcohol dehydrogenase (EC 1.1.1.1)).

Sialidases. The original species description of *D. pigrum* reports production of acid from D-glucose, galactose, D-fructose, D-mannose, maltose and L-fucose in two isolates with strain-level variation in producing acid from D-arabinose, mannitol, sucrose and N-acetyl-glucosamine (4). Glucose is the main monosaccharide detected in the nasal environment (1). Host-cell-surface- and host-mucin-derived sialic acids are another important potential source of monosaccharides and all 11 *D. pigrum* genomes harbor a putative sialidase as

well as a predicted transporter (sodium solute symporter) and catabolic enzymes suggesting it utilizes sialic acid in the nasal passages.

III. *D. pigrum* is predicted to be broadly susceptible to antibiotics and lacks virulence factors

Antibiotic resistance prediction. We analyzed all 11 *D. pigrum* genomes for putative antibiotic resistance genes and mutations that confer antibiotic resistance using the Resistance Gene Identifier (RGI) on the Comprehensive Antibiotic Resistance Database (CARD) (5) allowing only perfect and strict results. This analysis identified a candidate in only one strain. These data are consistent with the report from LaClaire and Facklam of *D. pigrum* susceptibility to amoxicillin, cefotaxime, cefuroxime, clindamycin, levofloxacin, meropenem, penicillin, quinupristin-dalfopristin, rifampin, tetracycline, and vancomycin for all tested *D. pigrum* strains (6). *D. pigrum* strain CDC 4709-98 alone encoded a predicted bleomycin resistance protein (BRP), which was predicted with 100% amino acid in a protein homolog model. In agreement, ResFinder (7) identified kanamycin nucleotidyltransferase (*aadD*, aka ANT(4')-Ia, aminoglycoside adenyltransferase AAD, spectinomycin resistance; streptomycin resistance; transferase) in this strain with 99.74 % sequence identity. Detailed analysis of this region with PlasmidFinder (8) and BLAST (9) revealed loci with sequence identity to portions of plasmid pUB110 from *S. aureus* (10) suggesting integration of, or at least part of, this plasmid into the genome of strain CDC 4709-98.

Virulence factor predictions. When compared to *S. aureus* or *Enterococcus species* using VirulenceFinder (11), the 11 *D. pigrum* genomes lacked predicted virulence factors.

Methods: Supplemental File S2

Biosynthetic gene clusters, antibiotic resistance genes and virulence factor genes. AntiSMASH (antibiotics & Secondary Metabolite Analysis SHell) and ClusterFinder (12, 13) were accessed at https://antismash.secondarymetabolites.org/ using default setpoints. We used antiSMASH version 5.0.0beta1-4e548fe (**Table A** and **Figure A**) (14). Putative antibiotic resistance genes or mutations in genes conferring antibiotic resistance were predicted using RGI on CARD (5). Assembly contigs were submitted at RGI (https://card.mcmaster.ca/analyze/rgi) and only perfect and strict hits were allowed. ResFinder version 2.1. (https://cge.cbs.dtu.dk/services/ResFinder/) with 90% threshold for %ID and 60% minimum length (7). We searched the 11 *D. pigrum* for predicted virulence factors using VirulenceFinder 2.0 (software version 2020-05-21; database version 2020-05-29; https://cge.cbs.dtu.dk/services/VirulenceFinder/) comparing each to both *S. aureus* and to *Enterococcus* species (threshold for %ID = 90; minimum length = 60%) (11).

Table A. Predicted biosynthetic gene clusters / secondary metabolites regions.

Strain	Bacteriocins	Lanthipeptides
KPL1914	2	1
39-95	1	1
2949-98	1	0
4294-98	2	0
4420-98	0	0
4545-98	1	0
4709-98	2	1
4199-99	3	0
4791-99	0	0
4792-99	2	0
ATCC51524	0	2

Figure A. Predicted biosynthetic gene clusters / secondary metabolites regions. Screen shot of the results for the antiSMASH analysis of the *D. pigrum* strains showing the type and position of each of the predicted biosynthetic gene clusters (14).

KPL1914

Region	Type	From	To	Most similar known cluster	Similarity	MIBiG BGC-ID
The following regions are from record 1:						
Region 1	Bacteriocin	166955	178641			
Region 2	Lanthipeptide	459577	487064			
Region 3	Bacteriocin	1238792	1250948			

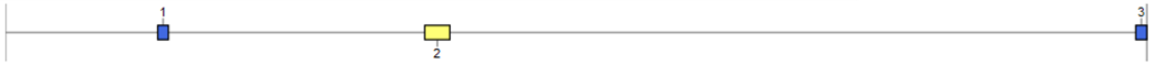

CDC4709-98

Region	Type	From	To	Most similar known cluster	Similarity	MIBiG BGC-ID
The following regions are from record 1:						
Region 1	Lanthipeptide	418481	441708			
Region 2	Bacteriocin	536284	545549			
Region 3	Bacteriocin	1239084	1249380			

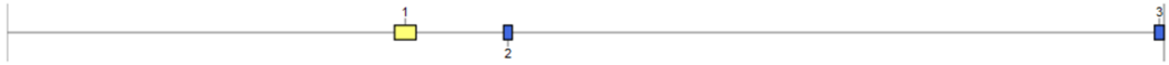

CDC39-95

Region	Type	From	To	Most similar known cluster	Similarity	MIBiG BGC-ID
The following regions are from record c00009_NODE_10.. (original name was: NODE_10_length_97139_cov_86.161758):						
Region 9.1	Bacteriocin	83002	95155	Non-acetylated open-chain sophorolipid / diacetylated lactonic other	100%	BGC0001274
The following regions are from record c00017_NODE_75.. (original name was: NODE_75_length_77128_cov_66.578545):						
Region 17.1	Lanthipeptide	41076	63532			

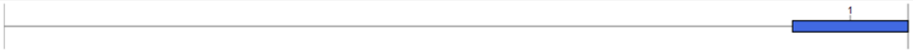

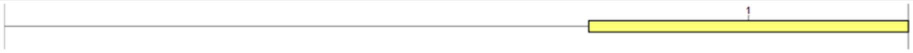

CDC2949-98

Region	Type	From	To	Most similar known cluster	Similarity	MIBiG BGC-ID
The following regions are from record c00036_NODE_16.. (original name was: NODE_165_length_169774_cov_66.562698):						
Region 36.1	Bacteriocin	132959	145112			

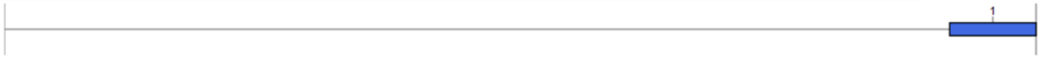

CDC4294-98

Region	Type	From	To	Most similar known cluster	Similarity	MIBiG BGC-ID
The following regions are from record c00014_NODE_14.. (original name was: NODE_14_length_25558_cov_121.382584):						

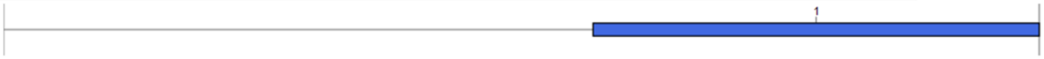

Region	Type	From	To	Most similar known cluster	Similarity	MIBiG BGC-ID
Region 14.1	Bacteriocin	14627	25696			
The following regions are from record c00028_NODE_36.. (original name was: NODE_36_length_49203_cov_111.674515):						

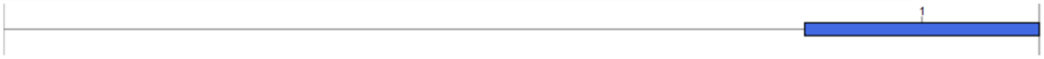

Region	Type	From	To	Most similar known cluster	Similarity	MIBiG BGC-ID
Region 28.1	Bacteriocin	38167	49341			

CDC4545-98

Region	Type	From	To	Most similar known cluster	Similarity	MIBiG BGC-ID
The following regions are from record c00010_NODE_30.. (original name was: NODE_30_length_491949_cov_138.342133):						

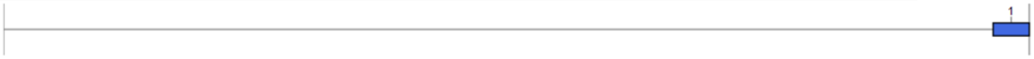

Region	Type	From	To	Most similar known cluster	Similarity	MIBiG BGC-ID
Region 10.1	Bacteriocin	333003	345156			

CDC4199-99

Region	Type	From	To	Most similar known cluster	Similarity	MIBiG BGC-ID
The following regions are from record c00016_NODE_52.. (original name was: NODE_52_length_379674_cov_119.267921):						

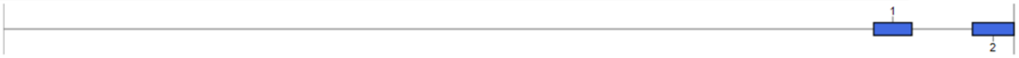

Region	Type	From	To	Most similar known cluster	Similarity	MIBiG BGC-ID
Region 16.1	Bacteriocin	254448	265629			
Region 16.2	Bacteriocin	283318	295471			
The following regions are from record c00030_NODE_10.. (original name was: NODE_100_length_155311_cov_111.723755):						

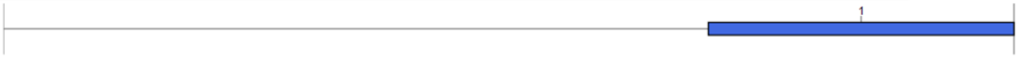

Region	Type	From	To	Most similar known cluster	Similarity	MIBiG BGC-ID
Region 30.1	Bacteriocin	23742	34038			

CDC4792-99

Region	Type	From	To	Most similar known cluster	Similarity	MIBiG BGC-ID
The following regions are from record c00013_NODE_48.. (original name was: NODE_48_length_252929_cov_101.571663):						

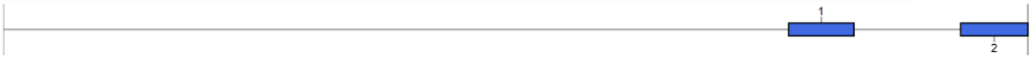

Region	Type	From	To	Most similar known cluster	Similarity	MIBiG BGC-ID
Region 13.1	Bacteriocin	141759	153571			
Region 13.2	Bacteriocin	172804	184957			

ATCC51524

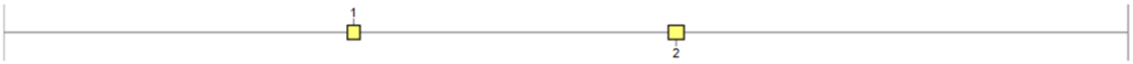

Region	Type	From	To	Most similar known cluster	Similarity
Region 1	lanthipeptide	564,388	585,116		
Region 2	lanthipeptide	1,091,260	1,117,015		

References: Supplemental File S2

1. Krismer B, Liebeke M, Janek D, Nega M, Rautenberg M, Hornig G, Unger C, Weidenmaier C, Lalk M, Peschel A. 2014. Nutrient limitation governs *Staphylococcus aureus* metabolism and niche adaptation in the human nose. PLoS Pathog 10:e1003862.

2. Krismer B, Weidenmaier C, Zipperer A, Peschel A. 2017. The commensal lifestyle of *Staphylococcus aureus* and its interactions with the nasal microbiota. Nat Rev Microbiol 15:675-687.

3. Buyze G, Van Den Hamer CJ, De Haan PG. 1957. Correlation between hexosemonophosphate shunt, glycolytic system and fermentation-type in Lactobacilli. Antonie Van Leeuwenhoek 23:345-50.

4. Aguirre M, Morrison D, Cookson BD, Gay FW, Collins MD. 1993. Phenotypic and phylogenetic characterization of some *Gemella*-like organisms from human infections: description of *Dolosigranulum pigrum* gen. nov., sp. nov. J Appl Bacteriol 75:608-12.

5. McArthur AG, Waglechner N, Nizam F, Yan A, Azad MA, Baylay AJ, Bhullar K, Canova MJ, De Pascale G, Ejim L, Kalan L, King AM, Koteva K, Morar M, Mulvey MR, O'Brien JS, Pawlowski AC, Piddock LJ, Spanogiannopoulos P, Sutherland AD, Tang I, Taylor PL, Thaker M, Wang W, Yan M, Yu T, Wright GD. 2013. The comprehensive antibiotic resistance database. Antimicrob Agents Chemother 57:3348-57.

6. Laclaire L, Facklam R. 2000. Antimicrobial susceptibility and clinical sources of *Dolosigranulum pigrum* cultures. Antimicrob Agents Chemother 44:2001-3.

7. Zankari E, Hasman H, Cosentino S, Vestergaard M, Rasmussen S, Lund O, Aarestrup FM, Larsen MV. 2012. Identification of acquired antimicrobial resistance genes. J Antimicrob Chemother 67:2640-4.

8. Carattoli A, Zankari E, Garcia-Fernandez A, Voldby Larsen M, Lund O, Villa L, Moller Aarestrup F, Hasman H. 2014. In silico detection and typing of plasmids using PlasmidFinder and plasmid multilocus sequence typing. Antimicrob Agents Chemother 58:3895-903.

9. Altschul SF, Gish W, Miller W, Myers EW, Lipman DJ. 1990. Basic local alignment search tool. J Mol Biol 215:403-10.

10. McKenzie T, Hoshino T, Tanaka T, Sueoka N. 1986. The nucleotide sequence of pUB110: some salient features in relation to replication and its regulation. Plasmid 15:93-103.

11. Joensen KG, Scheutz F, Lund O, Hasman H, Kaas RS, Nielsen EM, Aarestrup FM. 2014. Real-time whole-genome sequencing for routine typing, surveillance, and outbreak detection of verotoxigenic *Escherichia coli*. J Clin Microbiol 52:1501-10.

12. Medema MH, Blin K, Cimermancic P, de Jager V, Zakrzewski P, Fischbach MA, Weber T, Takano E, Breitling R. 2011. antiSMASH: rapid identification, annotation and analysis of secondary metabolite biosynthesis gene clusters in bacterial and fungal genome sequences. Nucleic Acids Res 39:W339-46.

13. Weber T, Blin K, Duddela S, Krug D, Kim HU, Bruccoleri R, Lee SY, Fischbach MA, Muller R, Wohlleben W, Breitling R, Takano E, Medema MH. 2015. antiSMASH 3.0-a comprehensive resource for the genome mining of biosynthetic gene clusters. Nucleic Acids Res 43:W237-43.

14. Blin K, Shaw S, Steinke K, Villebro R, Ziemert N, Lee SY, Medema MH, Weber T. 2019. antiSMASH 5.0: updates to the secondary metabolite genome mining pipeline. Nucleic Acids Res doi:10.1093/nar/gkz310.
