# Supplementary material for: Dolosigranulum pigrum Cooperation and Competition in Human Nasal Microbiota
Source: mSphere. 2020 Sep 9;5(5):e00852-20. doi: 10.1128/mSphere.00852-20 (PMC7485692; doi:10.1128/mSphere.00852-20)
Supplement: TABLE S3 [file mSphere.00852-20-st003.docx]

**Table S3: Non-*D. pigrum* bacterial strains used in this study**

| **Species** | **Strain** | **Internal Reference** | **Reference** | **Characteristics** |
| --- | --- | --- | --- | --- |
| *C. accolens* | KPL1818 | KPL1818 | [1] | primary adult human nostril isolate; lipid dependent |
| *C. pseudodiphtheriticum* | KPL1989 | KPL1989 | This study | primary adult human nostril isolate;  lipid independent |
| *C. pseudodiphtheriticum* | DSM44287^T^ | KPL2589 | [2] | type strain;  lipid independent |
| *C. propinquum* | DSM44285^T^ | KPL1955 | [3] | type strain;  lipid independent |
| *S. aureus* | Newman | KPL2023 | [4] | lab-adapted strain |
| *S. aureus* | JE2 | KPL2115 | [5] | plasmid-free derivative of USA300 LAC |
| *S. pneumoniae* | TIGR4 | KPL1904 | [6] | Clinical isolate |
| *S. pneumoniae* | DBL5 | KPL1905 | [7] | Clinical isolate |
| *S. pneumoniae* | 603 | KPL1906 | [8] | Clinical isolate |
| *S. pneumoniae* | WU2 | KPL1907 | [9] | Clinical isolate |

**References**

1. Bomar L, Brugger SD, Yost BH, Davies SS, Lemon KP: **Corynebacterium accolens Releases Antipneumococcal Free Fatty Acids from Human Nostril and Skin Surface Triacylglycerols.** *MBio* 2016, **7:**e01725-01715.

2. Lehmann KB, Neumann RO: **Corynebacterium pseudodiphtheriticum.** In *Atlas und Grundriss der Bakteriologie und Lehrbuch der Speziellen Bakteriologischen Diagnostik.* 1920 edition. Munich: J.F. Lehmann; 1896: 571-572

3. Riegel P, de Briel D, Prevost G, Jehl F, Monteil H: **Proposal of *Corynebacterium propinquum* sp. nov. for *Corynebacterium* group ANF-3 strains.** *FEMS Microbiology Letters* 1993, **113:**229-234.

4. Miller KD, Hetrick DL, Bielefeldt DJ: **Production and properties of *Staphylococcus aureus* (strain Newman D2C) with uniform clumping factor activity.** *Thromb Res* 1977, **10:**203-211.

5. Fey PD, Endres JL, Yajjala VK, Widhelm TJ, Boissy RJ, Bose JL, Bayles KW: **A genetic resource for rapid and comprehensive phenotype screening of nonessential *Staphylococcus aureus* genes.** *MBio* 2013, **4:**e00537-00512.

6. Tettelin H, Nelson KE, Paulsen IT, Eisen JA, Read TD, Peterson S, Heidelberg J, DeBoy RT, Haft DH, Dodson RJ, et al: **Complete genome sequence of a virulent isolate of Streptococcus pneumoniae.** *Science* 2001, **293:**498-506.

7. Lu YJ, Leite L, Goncalves VM, Dias Wde O, Liberman C, Fratelli F, Alderson M, Tate A, Maisonneuve JF, Robertson G, et al: **GMP-grade pneumococcal whole-cell vaccine injected subcutaneously protects mice from nasopharyngeal colonization and fatal aspiration-sepsis.** *Vaccine* 2010, **28:**7468-7475.

8. Malley R, Lipsitch M, Stack A, Saladino R, Fleisher G, Pelton S, Thompson C, Briles D, Anderson P: **Intranasal immunization with killed unencapsulated whole cells prevents colonization and invasive disease by capsulated pneumococci.** *Infect Immun* 2001, **69:**4870-4873.

9. Briles DE, Nahm M, Schroer K, Davie J, Baker P, Kearney J, Barletta R: **Antiphosphocholine antibodies found in normal mouse serum are protective against intravenous infection with type 3 streptococcus pneumoniae.** *J Exp Med* 1981, **153:**694-705.
